# The ubiquitin-binding protein ANKRD13A mediates VCP-dependent mitochondrial outer membrane rupture during PINK1/Parkin-mediated mitophagy

**DOI:** 10.1016/j.jbc.2025.110739

**Published:** 2025-09-18

**Authors:** Wei-Hua Chu, Yu-Shan Lin, Jing Guo, Wann-Neng Jane, Won-Jing Wang, Yu-Yang Lin, Pei-Han Liu, Po-Yu Huang, Wei-Chung Chiang

**Affiliations:** 1 Program in Molecular Medicine, National Yang Ming Chiao Tung University and Academia Sinica, Taipei, Taiwan; 2 Institute of Biochemistry and Molecular Biology, College of Life Sciences, National Yang Ming Chiao Tung University, Taipei, Taiwan; 3 Institute of Plant and Microbial Biology, Academia Sinica, Taipei, Taiwan; 4 Cancer and Immunology Research Center, National Yang Ming Chiao Tung University, Taipei, Taiwan

**Keywords:** mitochondrial quality control, PINK1/Parkin-mediated mitophagy, mitochondrial outer membrane rupture, ANKRD13A, VCP

## Abstract

PINK1/Parkin-mediated mitophagy is a major homeostatic mechanism by which cells selectively remove damaged, depolarized mitochondria. A signature event in this form of mitophagy is the rupture of the mitochondrial outer membrane (OMM), a process required for proper disposal of mitochondria. The OMM rupture results in the topological exposure of the mitochondrial inner membrane (IMM) mitophagy receptors, which are recognized by autophagy machinery, thus promoting the turnover of the depolarized mitochondria. However, due to the lack of efficient tools to measure OMM rupture, our mechanistic understanding of this process has been limited. In this study, we identified ankyrin repeat domain-containing protein 13 A (ANKRD13A) as a novel mitophagy factor that interacts with multiple mitochondrial proteins and relocalizes to the depolarized mitochondria. ANKRD13A promotes PINK1/Parkin-mediated mitophagy by recruiting valosin-containing protein (VCP), an AAA-ATPase that functions to remodel protein complexes or membranes *via* the extraction of protein substrates. Through the development of a novel biosensor that fluorescently marks the sites of OMM rupture, we visualized the OMM rupture events *in cellulo* and revealed that VCP and its recruitment factors, including ANKRD13A, are required for the rupture of OMM. This finding demonstrated that VCP-dependent remodeling of OMM during PINK1/Parkin-mediated mitophagy is a key driving force behind the OMM rupture. Furthermore, our newly developed biosensor represents an effective, reliable method to detect OMM rupture during PINK1/Parkin-mediated mitophagy, and it is valuable for future mechanistic investigation of this process.

Mitochondria are energy-producing organelles that play a pivotal role in a wide range of fundamental cellular processes. Damaged mitochondria release reactive oxygen species and mitochondrial DNA, which elevate genotoxic stress and trigger inflammasome activation, consequently promoting tumorigenesis and aging (1, 2). Selective autophagy of mitochondria (also known as mitophagy) is a major homeostatic pathway by which eukaryotic cells remove damaged or superfluous mitochondria by lysosomal sequestration. Defects in mitophagy are linked to neurodegenerative disorders such as Parkinson’s disease, Alzheimer’s disease, and other age-related disorders (3, 4, 5, 6, 7). In line with this observation, mitochondrial functional impairment is also featured in these human diseases (8, 9, 10, 11). As such, the effective removal of damaged mitochondria is vital for maintaining cellular and organismal homeostasis.

On the damaged mitochondria, dissipation of mitochondrial membrane potential results in the accumulation and stabilization of PINK1 (a serine/threonine kinase), which phosphorylates the ubiquitin molecules that are basally associated with mitochondrial outer membrane (OMM) proteins. The phospho-ubiquitin activates and recruits E3 ubiquitin ligase Parkin to the mitochondria. Once activated, Parkin ubiquitylates the OMM proteins, adding more ubiquitin molecules that further serve as substrates for PINK1, resulting in a further increase in Parkin recruitment and activation. This feed-forward mechanism amplifies the initial mitophagy signals and ensures a robust response against acute mitochondrial damage. Following PINK1/Parkin activation, phospho-ubiquitin-decorated mitochondria are recognized by a set of ubiquitin-binding autophagy receptors that bind to LC3 (an autophagosomal membrane protein essential for cargo recognition). Additional autophagy-related (ATG) proteins are also recruited to promote the formation of autophagic membranes around the depolarized mitochondria. Studies in the field of PINK1/Parkin-dependent mitophagy research mainly focused on the events that specify substrate selectivity on the OMM, such as the stabilization of PINK1, mitochondrial recruitment of Parkin, ubiquitylation of OMM proteins, and the recruitment of ubiquitin-binding autophagy adaptors (12, 13, 14). However, other forms of cargo recognition in the context of PINK1/Parkin-dependent pathway were not thoroughly explored.

Previous studies identified that the OMM undergoes focal rupture in a proteasome- and Parkin-dependent manner during PINK/Parkin-mediated mitophagy (15, 16). The inhibition of OMM rupture with proteasome inhibitors impedes the clearance of the depolarized mitochondria (16, 17), suggesting that the OMM rupture is required for PINK/Parkin-mediated mitophagy. But why must the OMM rupture during mitophagy? One plausible explanation is that the membrane rupture facilitates the release or exposure of inner mitochondrial components necessary for mitophagy. Previous studies have shown that mitochondrial inner membrane (IMM) proteins, once topologically exposed, act as points of recognition for selective autophagy. Specifically, during PINK1/Parkin-mediated mitophagy, the IMM protein prohibitin 2 (PHB2) biochemically interacts with LC3-II, promoting the clearance of depolarized mitochondria. Mutations of the LC3-interacting region (LIR) of PHB2 disrupt the binding and block PINK1/Parkin-mediated mitophagy (17). Consistent with this, a study reported that mitochondrial stress or elevated AMFR (an E3 ubiquitin ligase) triggers a distinct form of OMM rupture that allows endoplasmic reticulum (ER) membrane protein RETREG1 (an ER-phagy receptor) to interact with IMM protein OPA1, hence promoting the selective autophagy of both ER and mitochondria (18). These discoveries revealed that the recognition of IMM proteins constitutes a key mechanism in PINK1/Parkin-mediated mitophagy. Moreover, they indicate that OMM rupture is not simply a consequence of mitochondrial degradation but a regulated step that confers substrate selectivity for mitophagic clearance. Despite these findings, our understanding of OMM rupture remains limited. The requirement of proteasome and Parkin for OMM rupture suggests that this is a regulated process. Yet, the molecular mechanisms behind the OMM break during PINK1/Parkin-mediated mitophagy and the molecular events associated with it are poorly understood.

## Results

### Proteomic identification of mitophagy-regulated PHB2 interactome

**Figure 1:**
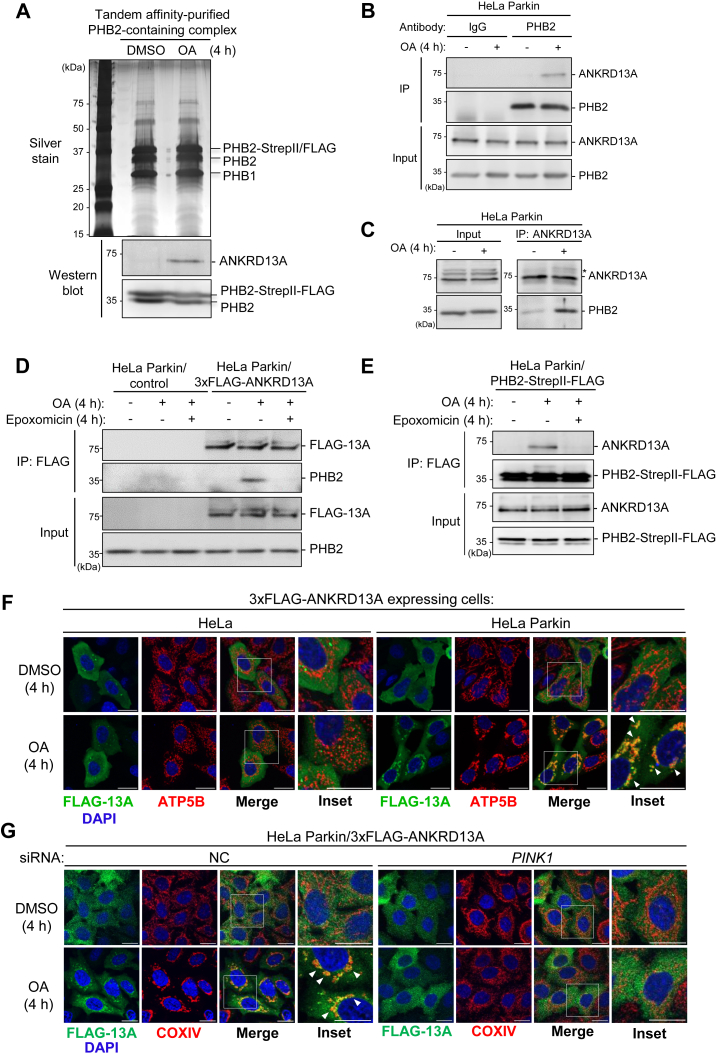

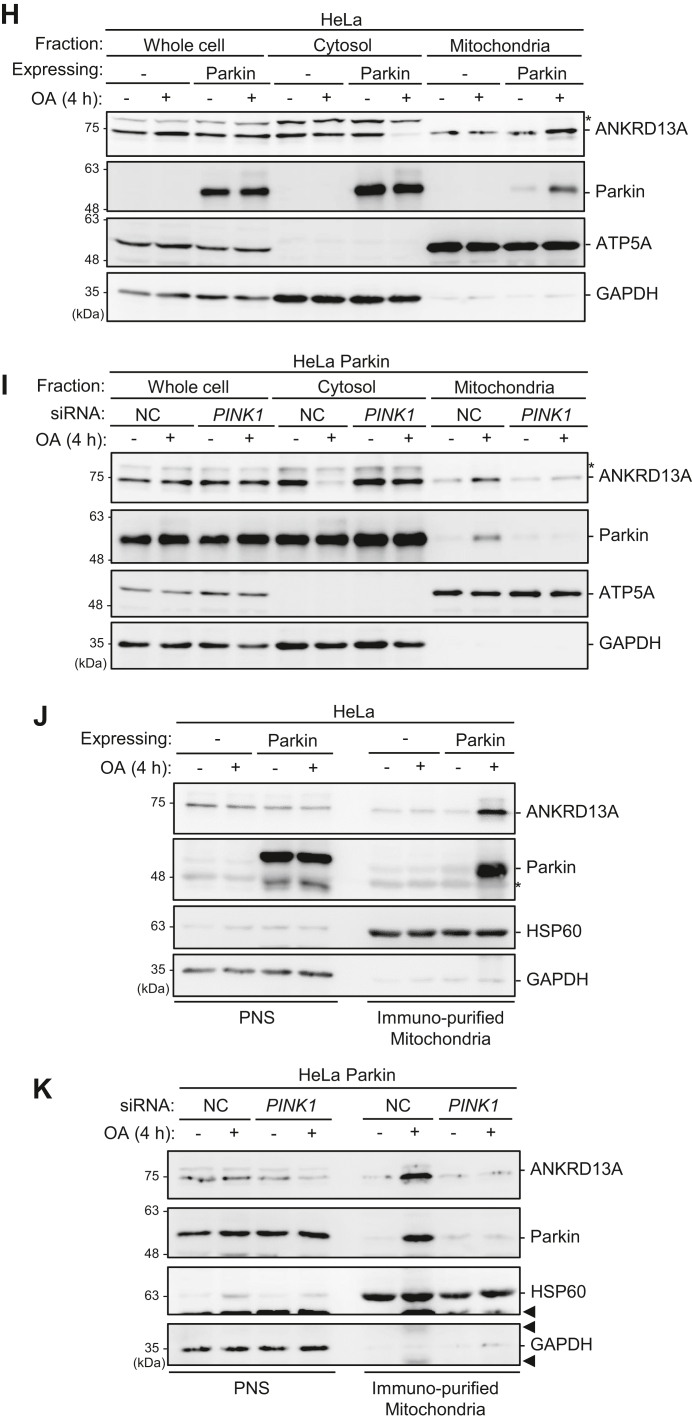
**ANKRD13A interacts with PHB2 and relocalizes to the mitochondria during PINK1/Parkin-mediated mitophagy.***A*, purification of PHB2-containing protein complexes by Strep-Tactin resin followed by anti-FLAG pull-down in HeLa Parkin cells stably expressing PHB2-StrepII-FLAG. Cells were treated with either DMSO or OA (2.5 μM oligomycin; 250 nM antimycin A for 4 h prior to the proteomic analysis. *B*-*C*, Co-immunoprecipitation of endogenous PHB2 and ANKRD13A in HeLa Parkin cells. Cells were treated with either DMSO or OA for 4 h prior to the immunoprecipitation with either anti-PHB2 or anti-ANKRD13A antibodies. *Asterisks* denote nonspecific bands. *D*-*E*, co-immunoprecipitation of PHB2 with ANKRD13A in HeLa Parkin cells expressing either 3xFLAG-ANKRD13A (FLAG-13A) or PHB2-StrepII-FLAG. Cells were treated with either DMSO, OA, or OA + epoxomicin (200 nM) for 4 h prior to the immunoprecipitation with anti-FLAG antibody. *F*, OA-induced mitochondrial relocalization of ANKRD13A is Parkin-dependent. HeLa or HeLa Parkin cells expressing 3xFLAG-ANKRD13A were treated with either DMSO or OA for 4 h and immunofluorescently stained for COXIV (mitochondrial marker) and FLAG-tagged ANKRD13A (FLAG-13A). *Arrows* denote the mitochondria encapsulated in ANKRD13A-positive structures. The scale bars represent 20 μm. *G*, OA-induced mitochondrial relocalization of FLAG-tagged ANKRD13A requires PINK1. HeLa Parkin cells treated either control (NC) or *PINK1* siRNA were incubated and stained as in (*F*). The scale bars represent 20 μm. *H*, Western blot analysis of cellular fractions isolated by differential centrifugation. HeLa or HeLa Parkin cells were treated with either DMSO or OA for 4 h prior to fractionation. *I*, Western blot analysis of cellular fractions from HeLa Parkin cells treated with either control (NC) or *PINK1* siRNA, and then incubated as in (*H*) prior to fractionation. *J*, Western blot analysis of immunopurified mitochondria from HeLa and HeLa Parkin cells treated with either DMSO or OA for 4 h. *K*, Western blot analysis of immunopurified mitochondria from HeLa Parkin cells treated with control (NC) or *PINK1* siRNA and followed by an incubation as in (*J*). PNS, post-nuclear supernatant. HSP60 and COXIV, mitochondrial markers. GAPDH, cytosol marker. The *arrows* indicate the bands in the prior blot. *Asterisks* denote nonspecific bands. ANKRD13A, ankyrin repeat domain-containing protein 13 A; DMSO, dimethyl sulfoxide; OA, oligomycin and antimycin; PHB1, prohibitin 1; PHB2, prohibitin 2.

To investigate the molecular events associated with OMM rupture during PINK1/Parkin-mediated mitophagy, we focused on PHB2, an IMM mitophagy receptor. Since PHB2 is topologically exposed during OMM rupture, we hypothesized that during PINK1/Parkin-mediated mitophagy, proteins recruited to the OMM rupture site could associate with PHB2. We conducted a proteomic screen to identify PHB2-interacting proteins, specifically those whose interactions with PHB2 are increased during PINK1/Parkin-mediated mitophagy. To do this, we stably expressed Parkin and tandem affinity-tagged PHB2 (PHB2-StrepII-FLAG) in HeLa cells. We affinity purified PHB2-containing protein complex from cells treated with either dimethyl sulfoxide (DMSO) or oligomycin and antimycin (OA, mitochondria depolarizing agents) for 4 h (Fig. 1*A*, top). This duration was chosen because mitochondrial clearance is not evident at this time point. Label-free complex mixture analyses with mass spectrometry identified a consistent interaction with PHB1, a protein that forms prohibitin complex with PHB2 on the IMM, in both basal and mitophagy-inducing conditions (Table 1), indicating that the formation of prohibitin complex is not affected by mitophagy induction with OA. Despite the overall low spectra counts, several unique proteins were found to be enriched in OA-treated samples, suggesting that PHB2 interacts with specific proteins upon OA-induced mitophagy.

**Table 1:** Identification of mitophagy-regulated PHB2-interacting proteins

Protein	Description	# Unique peptide	% Coverage	Spectra counts		Normalized spectral index (MIC Sin × 10^−8^)		OA/DMSO ratio
				DMSO	OA	DMSO	OA	
P35232	PHB_HUMAN Prohibitin	35	99.60	1250.80	1445.10	1540	1590	1.03
Q99623	PHB2_HUMAN Prohibitin-2	44	87.00	1507.10	1655.93	1770	1720	0.97
B4DV12	B4DV12_HUMAN Ubiquitin	10	79.70	17.72	57.18	105.7	2193	20.75
O60260	PRKN2_HUMAN E3 ubiquitin-protein ligase Parkin	15	46.20	0	19.75	0	19.33	OA Only
Q8IZ07	AN13A_HUMAN Ankyrin repeat domain-containing protein 13A	3	7.10	0	6.00	0	2.69	OA Only
P52597	HNRPF_HUMAN Heterogeneous nuclear ribonucleoprotein F	7	21.90	0	3	0	1.80	OA Only

HeLa Parkin cells expressing PHB2-StrepII-FLAG were treated with either DMSO or OA for 4 h prior to protein isolation. PHB2-containing protein complexes were tandem affinity-purified and analyzed by mass spectrometry. The normalized spectral index (which takes account of both protein length and the number of unique peptides per protein) indicates the relative abundance of the candidate proteins in DMSO- or OA-treated samples. OA/DMSO ratio indicates the ratio of normalized spectral index of OA- and DMSO-treated samples.

We found that ubiquitin is enriched in PHB2-containing protein complexes under mitophagy-inducing conditions (Table 1), suggesting that PHB2 or its interacting proteins may undergo ubiquitylation during PINK1/Parkin-mediated mitophagy. In addition, Parkin is also enriched in PHB2-containing protein complexes from OA-treated samples, indicating a mitophagy-induced interaction between Parkin and PHB2. This finding is in line with a recent report in which Parkin was found to bind to and ubiquitylate PHB2 (19). Furthermore, the proteomic analysis identified a molecule known as ankyrin repeat domain-containing protein 13 A (ANKRD13A) only in PHB2-containing protein complexes isolated from OA-treated cells. Western blot analysis of the affinity-isolated PHB2-containing protein complexes confirmed the interaction of ANKRD13A and PHB2 in the OA-treated sample (Fig. 1*A*, bottom). ANKRD13A is previously reported to mediate endolysosomal trafficking of caveolin-1 (20) and epidermal growth factor receptor (21) and regulate tumor necrosis factor signaling *via* binding to RIP1 (22), but its role in PINK1/Parkin-mediated mitophagy has not been previously reported.

### ANKRD13A interacts with PHB2 and relocalizes to the mitochondria during PINK1/Parkin-mediated mitophagy

Reciprocal co-immunoprecipitation in HeLa Parkin cells revealed that OA treatment induced an interaction between ANKRD13A and PHB2 at endogenous levels (Fig. 1, *B* and *C*) as well as upon exogenous expression of epitope-tagged ANKRD13A or PHB2 (Fig. 1, *D* and *E*). In addition, inhibition of OMM rupture using epoxomicin, a potent proteasome inhibitor, effectively abolished the OA-induced interaction between ANKRD13A and PHB2 (Fig. 1, *D* and *E*), indicating that ANKRD13A interacts with PHB2 on the IMM in a manner that requires proteasome-dependent OMM rupture.

The interaction between PHB2 and ANKRD13A upon OA treatment suggests that ANKRD13A is recruited to the depolarized mitochondria. The immunofluorescent analysis of HeLa Parkin cells expressing 3xFLAG-ANKRD13A showed that under basal conditions, ANKRD13A is primarily localized to the cytosol (Fig. 1*F*, top-right panel) but occasionally forms punctate structures that have been previously identified as endosomes (20). Following OA treatment, ANKRD13A is relocalized to the mitochondria (Fig. 1*F*, bottom-right panel). In HeLa cells, which lack endogenous Parkin expression, the relocalization of ANKRD13A during OA-induced mitophagy did not occur (Fig. 1*F*, left), suggesting that the mitochondrial recruitment of ANKRD13A occurs downstream of Parkin activation. Consistent with this, the depletion of PINK1 with siRNA completely blocked OA-induced mitochondrial recruitment of ANKRD13A (Fig. 1*G*). Thus, we conclude that ANKRD13A is relocalized to the depolarized mitochondria in a PINK1- and Parkin-dependent manner.

Due to the limited sensitivity of the anti-ANKRD13A antibody, we were not able to immunofluorescently assess the mitochondrial localization of ANKRD13A at endogenous levels. Instead, we performed differential centrifugation to isolate mitochondrial and cytosolic fractions. Western blot analyses showed that the treatment with OA led to the accumulation of ANKRD13A in the mitochondrial fraction, with a concomitant decrease in the cytosolic fraction in HeLa Parkin cells. This redistribution was not observed in cells lacking Parkin (Fig. 1*H*) or PINK1 (Fig. 1*I*), indicating that mitochondrial recruitment of ANKRD13A requires both proteins. To further validate these findings, we performed immunopurification of highly enriched mitochondria from post-nuclear supernatant (PNS) using anti-TOMM20 antibodies. Consistent with the fractionation data, Western blot analysis revealed a marked increase of mitochondrial ANKRD13A following OA-induced mitophagy in a PINK1- and Parkin-dependent manner (Fig. 1, *J* and *K*). Collectively, these results clearly demonstrated that ANKRD13A interacts with PHB2 and undergoes mitochondrial recruitment upon the activation of PINK1/Parkin-mediated mitophagy.

### ANKRD13A is required for mitophagy

**Figure 2:**
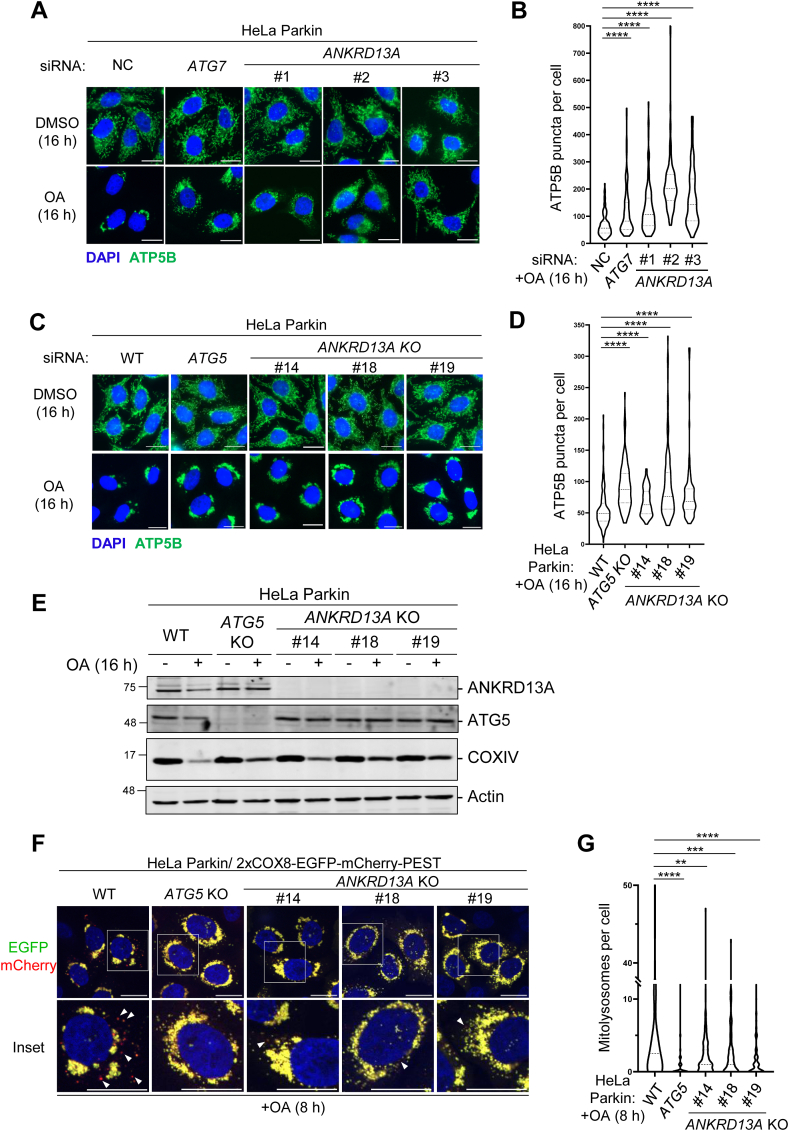

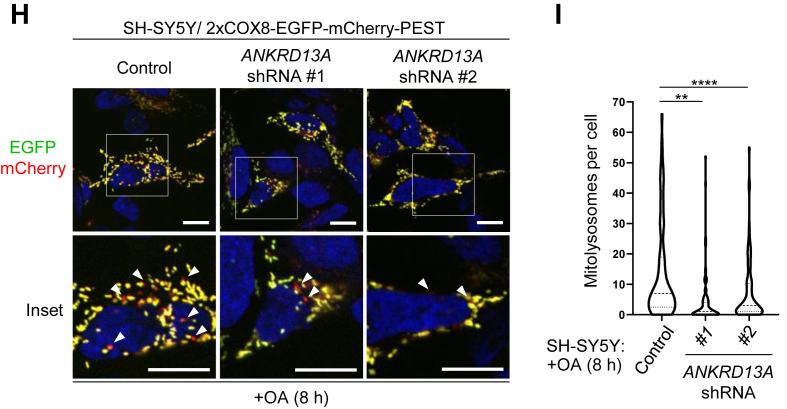
**ANKRD13A is required for PINK1/Parkin-mediated mitophagy.***A*, ANKRD13A is required for OA-induced mitochondrial clearance. Representative images of immunofluorescent staining of cytoplasmic ATP5B (mitochondrial marker) puncta. HeLa Parkin cells were treated with the indicated siRNA and subjected to either DMSO or OA treatment for 16 h. The scale bars represent 20 μm. *B*, quantitation of OA-induced clearance of ATP5B puncta in (*A*). At least 90 cells were analyzed per sample. The scale bars represent 20 μm. ∗∗∗∗*p* < 0.0001, one-way ANOVA with multiple comparisons. *C*, representative images of immunofluorescent staining of cytoplasmic ATP5B puncta in HeLa Parkin wild-type (WT) and *ANKRD13A* knockout (KO) cells treated with either DMSO or OA treatment for 16 h. The scale bars represent 20 μm. *D*, quantitation of OA-induced clearance of ATP5B puncta in wild-type (WT) and *ANKRD13A* KO HeLa Parkin cells treated as in (*C*). At least 100 cells were analyzed per sample. ∗∗∗∗*p* < 0.0001, one-way ANOVA with multiple comparisons. *E*, Western blot analysis of OA-induced clearance of mitochondrial protein COXIV in the indicated cell lines as treated in (*C*). *F*, mitophagy flux assay. Representative immunofluorescent micrographs of wild-type (WT) or *ANKRD13A* KO HeLa Parkin cells expressing mitophagy flux reporter (2xCOX8-EGFP-mCherry-PEST) incubated with either DMSO or OA for 8 h. *Arrows* indicate mitolysosomes (*red*). The scale bars represent 20 μm. *G*, quantitation of mitolysosomes in (*F*). At least 100 cells were analyzed per sample. ∗∗*p* < 0.01, ∗∗∗*p* < 0.001, ∗∗∗∗*p* < 0.0001, one-way ANOVA with multiple comparisons. *H*, representative immunofluorescent images of SH-SY5Y cells stably expressing control or *ANKRD13A*-targeting shRNA and the mitophagy flux reporter. Cells were incubated with either DMSO or OA for 8 h and analyzed. *Arrows* indicate mitolysosomes (*red*). The scale bars represent 10 μm. *I*, quantitation of mitolysosomes in (*H*). At least 75 cells were analyzed per sample. ∗∗*p* < 0.01, ∗∗∗∗*p* < 0.0001, one-way ANOVA with multiple comparisons. ANKRD13A, ankyrin repeat domain-containing protein 13 A; DMSO, dimethyl sulfoxide; OA, oligomycin and antimycin.

Selective autophagy often requires the gathering of specific receptor or effector proteins to the cargo for autophagic clearance. Thus, the mitochondrial recruitment of ANKRD13A in response to OA treatment suggested that ANKRD13A may be required for PINK1/Parkin-mediated mitophagy. To test this, we treated HeLa Parkin cells with control (NC), *ATG7* (a core autophagy gene), or *ANKRD13A* siRNA and monitored OA-induced clearance of ATP5B (a mitochondria marker)-positive structures. The siRNA treatment efficiently depleted ATG7 and ANKRD13A (Fig. S1*A*), and resulted in the accumulation of depolarized mitochondria to an extent similar to ATG7 knockdown following OA treatment (Fig. 2, *A* and *B*). To rule out the possibility of off-target effects of siRNA, we generated *ANKRD13A*-knockout (KO) cells by using CRISPR/Cas9 approach (Fig. S1, *B* and *C*). Consistent with our siRNA experiments, *ANKRD13A* KO impeded the clearance of ATP5B puncta (Fig. 2, *C* and *D*) as well as COXIV (mitochondria marker) (Fig. 2*E*), indicating that ANKRD13A is required for the clearance of depolarized mitochondria.

To rigorously examine the role of ANKRD13A in PINK1/Parkin-mediated mitophagy, we utilize a tandem bifluorescent protein reporter (2xCOX8-EGFP-mCherry-PEST) to examine whether ANKRD13A is required for the lysosomal delivery of depolarized mitochondria, a process known as “mitophagy flux” (23, 24). In HeLa Parkin cells expressing 2xCOX8-EGFP-mCherry-PEST, the depletion of ANKRD13A resulted in a significantly reduced number of mitolysosomes (as indicated by the number of red-only puncta) (Fig. 2, *F* and *G*), suggesting that ANKRD13A is crucial for lysosomal delivery of depolarized mitochondria. Moreover, we also examine the role of ANKRD13A in OA-induced mitophagy in cells expressing endogenous Parkin. shRNA-mediated knockdown of ANKRD13A in neuroblastoma cell line SH-SY5Y expressing the mitophagy flux reporter resulted in a significantly reduced OA-induced mitophagy flux (Fig. 2, *H* and *I*), indicating that ANKRD13A is also important for mitophagy when Parkin is expressed at endogenous, physiologically relevant levels. Together, these findings demonstrated that ANKRD13A is a *bona fide* factor for Parkin/PINK1-dependent mitophagy.

During iron depletion, a distinct form of mitophagy is initiated by the HIF1α-dependent upregulation of OMM mitophagy receptors NIX and BNIP3, which directly interact with LC3 and promote mitochondrial clearance (25). We investigated whether ANKRD13A is also involved in this form of mitophagy. Mitophagy flux assays in wild-type and *ANKRD13A* KO cells showed that deferiprone (DFP) treatment resulted in robust mitophagy, but the mitophagy flux in *ANKRD13A* KO cells was not significantly different from the wild-type cells (Fig. S1, *D* and *E*), indicating that ANKRD13A is dispensable for iron depletion-induced, NIX/BNIP3-dependent mitophagy, supporting its specific role in the PINK1/Parkin-dependent pathway.

### The ubiquitin-binding motifs (UIMs) and ankyrin repeat (AR) are required for the mitophagy functions of ANKRD13A

**Figure 3:**
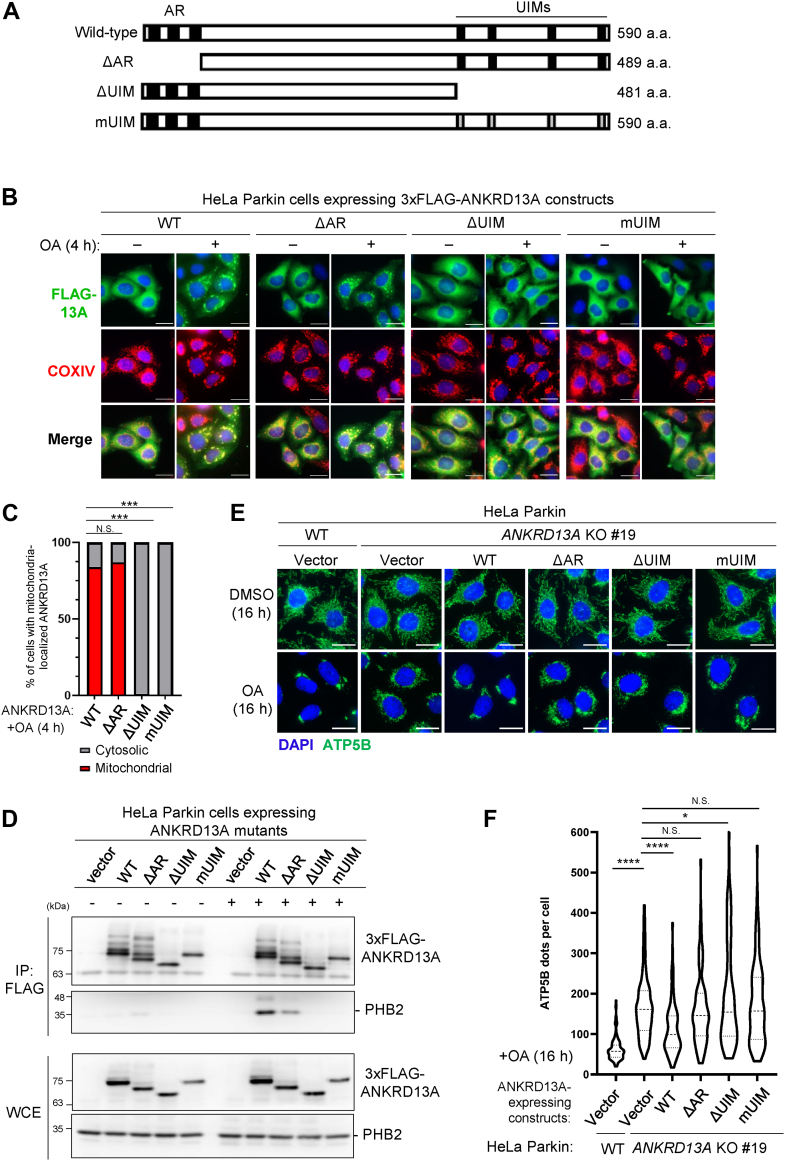

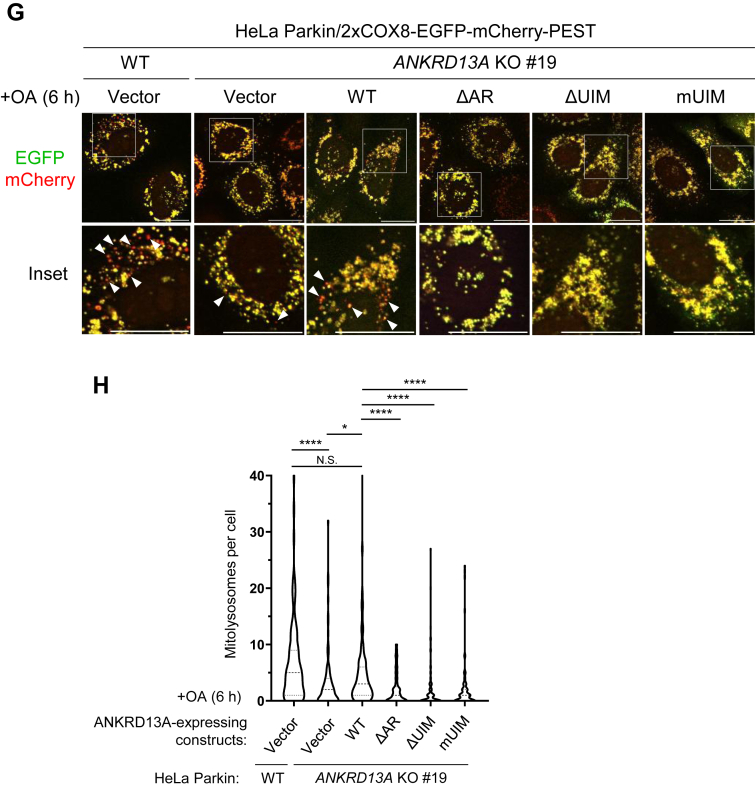
**The UIMs and AR domains are required for the mitophagy function of ANKRD13A.***A*, schematic diagram showing the domain structure of ANKRD13A and the mutants used in this study. WT: wild-type; ΔAR: deletion of N-terminal ankyrin repeat; ΔUIM: deletion of C-terminal region encompassing four ubiquitin-interacting motifs (UIMs); mUIM: mutation of key residues of four UIMs. *B*, representative micrographs showing the colocalization of 3xFLAG-tagged ANKRD13A (FLAG-13A) and the mitochondria (COXIV) in HeLa Parkin cells expressing wild-type (WT), ankyrin repeat deletion (ΔAR), UIM deletion (ΔUIM), and UIM mutant (mUIM) of 3xFLAG-ANKRD13A constructs. Samples were treated with either DMSO or OA for 4 h prior to the immunofluorescent analyses. The scale bars represent 20 μm. *C*, quantification of the samples shown in (*B*). At least 120 cells were analyzed per sample. N.S. non-significant, ∗∗∗*p* < 0.001, Chi-square test. The same experiments were repeated for at least three times. *D*, co-immunoprecipitation of PHB2 with ANKRD13A mutants. HeLa Parkin cells expressing wild-type or the indicated mutant of 3xFLAG-ANKRD13A were treated with either DMSO or OA for 4 h and immunoprecipitated with anti-FLAG antibody. Samples were analyzed by Western blot as indicated. *E*, representative images of immunofluorescent staining of ATP5B (mitochondrial marker) puncta in wild-type and *ANKRD13A* KO HeLa Parkin cells expressing empty vector (vector) or the indicated mutants of ANKRD13A. The samples were subjected to either DMSO or OA treatment for 16 h. The scale bars represent 20 μm. *F*, quantitation of ATP5B (mitochondrial marker) puncta in the sample shown in (*E*). At least 100 cells were analyzed per sample. ∗∗*p* < 0.01, ∗∗∗*p* < 0.001; one-way ANOVA with multiple comparisons. *G*, representative immunofluorescent images showing mitolysosomes in HeLa Parkin cells and *ANKRD13A* KO cells reconstituted with empty vector (vector) or mutants of ANKRD13A. Cells were transiently transfected with mitophagy flux reporter (2xCOX8-EGFP-mCherry-PEST) and incubated with either DMSO or OA for 6 h. *Arrows* indicate mitolysosomes (*red*). *H*, quantitation of mitolysosomes in (*G*). At least 80 cells were analyzed per sample. The scale bars represent 20 μm. N.S. non-significant, ∗*p* < 0.05, ∗∗∗∗*p* < 0.0001, one-way ANOVA with multiple comparisons. ANKRD13A, ankyrin repeat domain-containing protein 13 A; AR, ankyrin repeat; DMSO, dimethyl sulfoxide; OA, oligomycin and antimycin; PHB2, prohibitin 2.

To understand how ANKRD13A mediates PINK1/Parkin-mediated mitophagy, we next investigated the molecular determinants of the mitophagy function of ANKRD13A. Based on sequence homology, ANKRD13A has three putative N-terminal ankyrin repeats (ARs) with unknown function and four C-terminal ubiquitin-interacting motifs (UIMs) that were previously reported to recognize K63-linked ubiquitin chains (21). To identify the functional domain(s) of ANKRD13A that are necessary for its mitochondrial recruitment and PHB2 binding, we expressed FLAG-tagged ANKRD13A mutants with deletions or point mutations of the AR or UIMs (20) in HeLa Parkin cells (Fig. 3*A*). Immunofluorescent analyses revealed that either the removal (ΔUIM) or mutation of key residues responsible for ubiquitin-binding in the UIMs (mUIM) significantly diminished OA-induced mitochondrial recruitment of ANKRD13A (Fig. 3, *B* and *C*). The deletion of the AR (ΔAR) did not impact the mitochondrial localization of ANKRD13A; however, we observed that OA-induced perinuclear clustering of mitochondria is attenuated, indicating that the AR domain may also play a role in mitochondrial dynamics upon depolarization. Immunoprecipitation experiments revealed that both the mutation and deletion of the UIMs completely blocked the OA-induced interaction between ANKRD13A and PHB2 (Fig. 3*D*), while the deletion of the AR only partially diminished the interaction. Together, our data suggest that the UIMs of ANKRD13A are key determinants in both mitochondrial recruitment and PHB2 interaction of ANKRD13A during PINK1/Parkin-mediated mitophagy.

In addition, we also investigated the requirement of the AR and UIMs of ANKRD13A for OA-induced mitophagy. The wild-type, UIMs, or AR mutant of ANKRD13A was expressed in the *ANKRD13A* KO HeLa Parkin cells, and we monitored the clearance of the depolarized mitochondria following OA treatment. The *ANKRD13A* KO cells were unable to remove the depolarized mitochondria efficiently; however, such a defect in mitophagy can be rescued by the re-expression of wild-type, but not the UIM and AR mutants of ANKRD13A (Fig. 3, *E* and *F*). Consistent with this, mitophagy flux assays showed that the re-expression of wild-type ANKRD13A promoted the impaired mitophagy flux in *ANKRD13A* KO cells, but the UIM or AR mutants failed to do so. (Fig. 3, *G* and *H*). Thus, both the UIM and AR domains are essential for the mitophagy function of ANKRD13A.

### The interaction between PHB2 and ANKRD13A is ubiquitin-dependent

**Figure 4:**
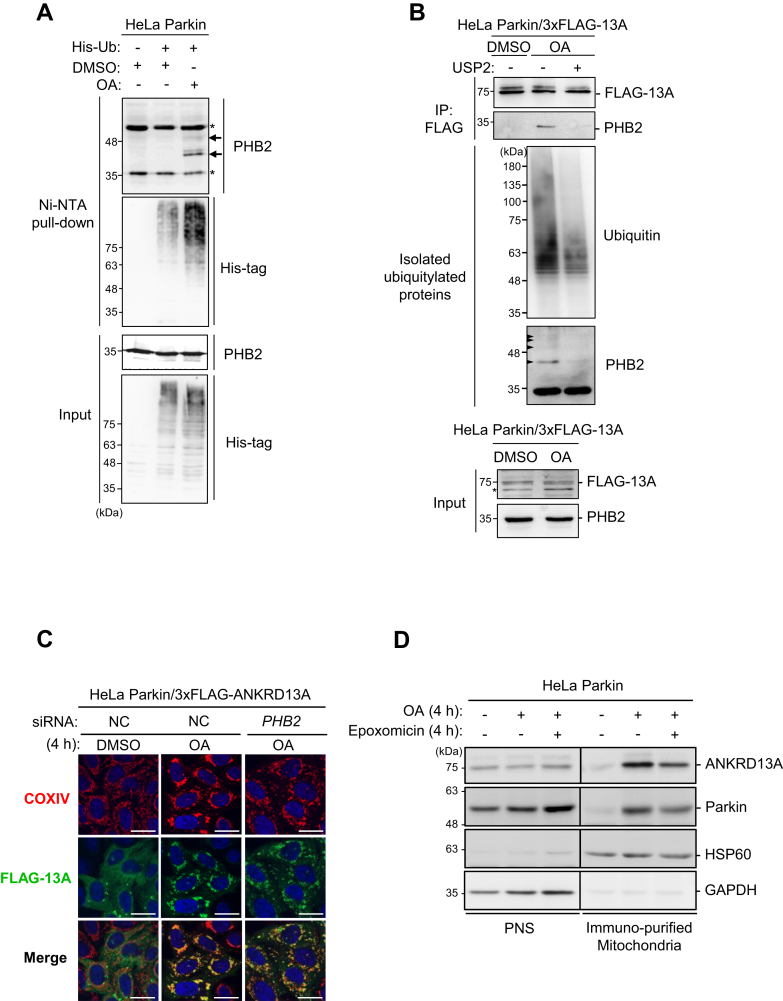

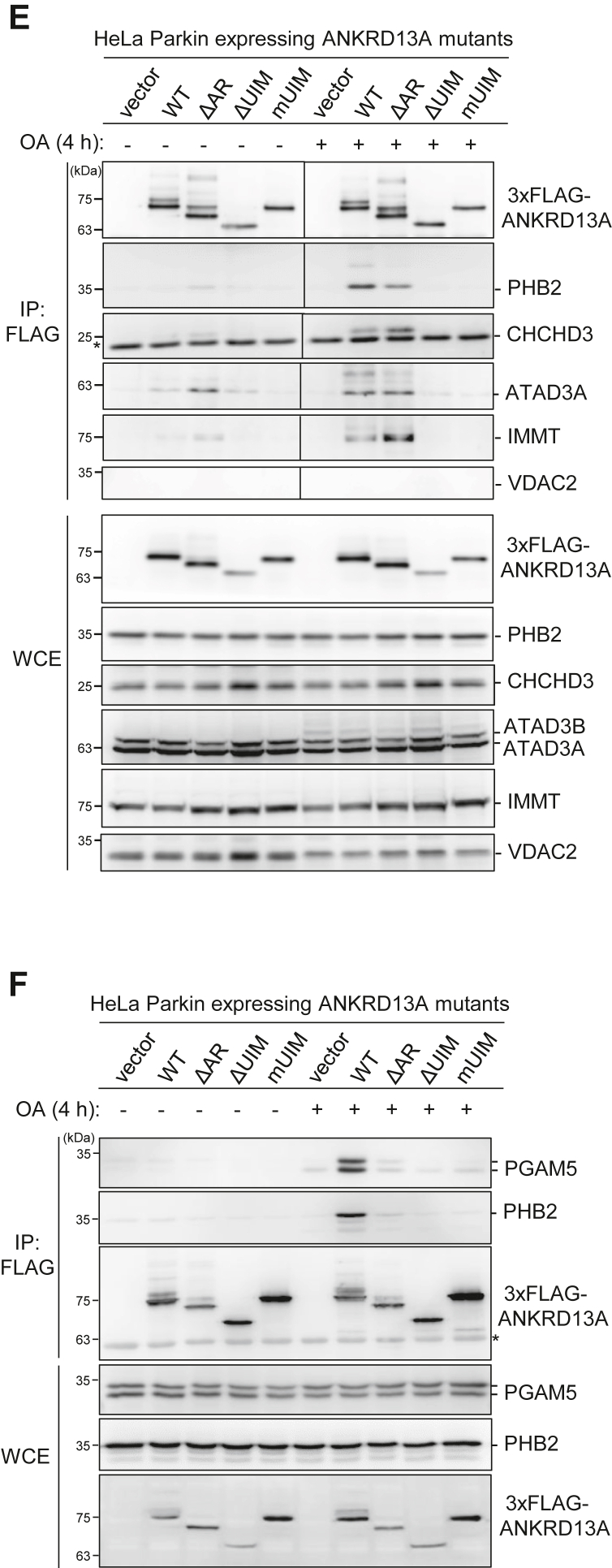
**ANKRD13A interacts with multiple mitochondrial proteins during OA-induced mitophagy in a UIM-dependent manner.***A*, HeLa Parkin cells transiently transfected with either empty vector or 6xHis-tagged ubiquitin (His-Ub)-expressing construct were subjected to either DMSO or OA treatment for 4 h prior to the purification of 6xHis-ubiquitylated proteins with Ni-NTA resin under denatured conditions. Samples were analyzed by Western blot as indicated. *Arrows* indicate ubiquitin-conjugated PHB2. *Asterisks* denote nonspecific bands. *B*, USP2 deubiquitinase assay. *Top panel*: HeLa Parkin cells expressing 3xFLAG-ANKRD13A were treated with either DMSO or OA for 4 h and immunoprecipitated with anti-FLAG antibody. The immunoprecipitates were incubated with or without purified recombinant catalytic domain of USP2 for 40 min and followed by Western blot analysis. *Middle panel*: isolated ubiquitylated proteins from OA-treated HeLa Parkin cells were incubated with USP2 for 40 min and Western blot analyzed as indicated. Incubation with purified USP2 effectively reduced ubiquitylated PHB2. The *arrows* indicate the ubiquitylated forms of PHB2. *Lower panel*: cell lysate input. *C*, HeLa Parkin cells expressing 3xFLAG-ANKRD13A were treated with either control (NC) or *PHB2* targeting siRNA and followed by treatment with either DMSO or OA for 4 h. Samples were immunofluorescently stained as indicated. The scale bars represent 20 μm. *D*, Western blot analysis of immunopurified mitochondria from HeLa Parkin cells treated with either DMSO, OA, or OA + epoxomicin (200 nM) for 4 h. *E*-*F*, HeLa Parkin expressing wild-type (WT), ankyrin repeat deletion (ΔAR), UIM deletion (ΔUIM), and UIM mutant (mUIM) of 3xFLAG-ANKRD13A were treated with either DMSO or OA for 4 h. Samples were immunoprecipitated with anti-FLAG antibody and analyzed by Western blot as indicated. *Asterisks* denote nonspecific bands. ANKRD13A, ankyrin repeat domain-containing protein 13 A; AR, ankyrin repeat; DMSO, dimethyl sulfoxide; OA, oligomycin and antimycin; PHB2, prohibitin 2; UIM, ubiquitin-interacting motif.

The UIMs of ANKRD13A are required for its interaction with PHB2 (Fig. 3*D*), suggesting that ANKRD13A binds to PHB2 through the recognition of ubiquitin. Our proteomic studies showed that ubiquitin is highly enriched in the PHB2-containing protein complex in response to OA treatment, indicating that PHB2 may undergo ubiquitylation during PINK1/Parkin-mediated mitophagy. Thus, we hypothesized that ANKRD13A recognizes the ubiquitylated form of PHB2 during mitophagy. To test this, we isolated ubiquitylated proteins from HeLa Parkin cells expressing 6x-His-ubiquitin under basal or mitophagy-inducing conditions. Western blot detection of ubiquitylated proteins revealed that OA treatment results in an increased PHB2 ubiquitylation in HeLa Parkin cells (Fig. 4*A*), confirming that PHB2 undergoes OA-induced ubiquitylation. This finding is consistent with a previous study in which Parkin-dependent PHB2 ubiquitylation sites (19, 26) were identified.

To investigate whether the interaction between ANKRD13A and PHB2 is ubiquitin-dependent, we performed deubiquitinase assays in which isolated ANKRD13A-containing protein complexes from HeLa Parkin cells were incubated with the purified recombinant catalytic domain of deubiquitinase USP2 (258–605 a.a.). USP2 effectively removed isolated ubiquitylated total proteins as well as the ubiquitinated forms of PHB2 (Fig. 4*B*, middle). Notably, treatment of FLAG-tagged ANKRD13A immunoprecipitants with USP2 markedly reduced the OA-induced interaction between ANKRD13A and PHB2 (Fig. 4*B*, top), indicating that their interaction is ubiquitin-dependent.

### Multiple mitochondrial proteins interact with ANKRD13A during PINK1/Parkin-mediated mitophagy

To examine whether the interaction of ANKRD13A and PHB2 is important for the mitochondrial recruitment of ANKRD13A during PINK1/Parkin-mediated mitophagy, we performed siRNA-mediated knockdown of PHB2 in HeLa Parkin cells and monitored OA-induced mitochondrial recruitment of ANKRD13A. The depletion of PHB2 resulted in fragmented, dispersed mitochondria that do not undergo perinuclear clustering upon OA treatment, which is in line with a previous report (17). However, the loss of PHB2 did not prevent OA-induced mitochondrial recruitment of ANKRD13A (Fig. 4*C*), suggesting that ANKRD13A recruitment may require interactions with additional mitochondrial proteins. Supporting this hypothesis, Western blot analysis of the immunopurified mitochondria showed that the OA-induced mitochondrial relocalization of ANKRD13A was only partially attenuated even when the proteasome-dependent OMM rupture is blocked by epoxomicin (Fig. 4*D*), indicating that ANKRD13A may associate with additional mitochondrial proteins on the OMM upon its recruitment.

To identify additional interacting partners of ANKRD13A during PINK1/Parkin-mediated mitophagy, we performed proteomic analysis of affinity-isolated ANKRD13A-containing protein complex from HeLa Parkin cells expressing Flag-tagged ANKRD13A. Label-free quantification revealed that mitochondrial proteins PHB1, PHB2, ATAD3A, IMMT, CHCHD3, PGAM5, and VDAC2 are highly enriched in OA-treated samples (Table 2). Co-immunoprecipitation showed that the binding of ANKRD13A to PHB2, ATAD3A, IMMT, CHCHD3, and PGAM5 was markedly increased upon mitophagy induction with OA, whereas VDAC2 did not interact with ANKRD13A at all (Fig. 4, *E* and *F*). The mutations or deletion of the UIMs abolished these interactions, while the deletion of the AR had no significant effect. These results suggested that during OA-induced mitophagy, ANKRD13A associates with multiple mitochondrial proteins, including PHB2, ATAD3A, IMMT, CHCHD3, and PGAM5, in a UIM-dependent manner, and the interaction with these proteins may be required for the recruitment of ANKRD13A. We attempted siRNA knockdown of either ATAD3, CHCHD3, IMMT, or PGAM5, but this did not abolish ANKRD13A recruitment (data not shown). We posit that the interaction of ANKRD13A with these mitochondrial proteins may collectively contribute to the recruitment of ANKRD13A during PINK1/Parkin-mediated mitophagy. However, we were unable to confirm this as treatment with multiple siRNAs did not result in effective simultaneous gene knockdown.

**Table 2:** Proteomic identification of ANKRD13A binding partners during PINK1/Parkin-mediated mitophagy

Protein	Gene name	# Unique peptide	% Coverage	Normalized spectral index		OA/DMSO ratio
				OA	DMSO	
Voltage-dependent anion-selective channel protein 2	VDAC2	2	6.5	4955050	0	--
Prohibitin	PHB	4	13.6	11482000	160605	71.49
Prohibitin-2	PHB2	6	17.1	55378500	1191600	46.47
ATPase family AAA domain-containing protein 3A	ATAD3A	5	8.2	9557550	436795	21.88
MICOS complex subunit MIC60	IMMT	19	22.3	104150000	5070750	20.54
MICOS complex subunit MIC19	CHCHD3	6	22.4	27479500	2150850	12.78
Valosin-containing protein	VCP	10	12.7	4665750	11115600	0.42
Ankyrin repeat domain-containing protein 13A	ANKRD13A	26	44.1	995515000	1366050000	0.73
Ubiquitin C	UBC	6	67.7	3293850000	2185550000	1.51

Select ANKRD13A-interacting proteins identified by mass spectrometry analyses from HeLa Parkin cells expressing 3xFlag-ANKRD13A undergoing OA-induced mitophagy. The cells were incubated with either DMSO or OA 4 h and Flag-tagged ANKRD13A-containing protein complex was purified with anti-Flag antibody. The relative enrichment of the candidate proteins in DMSO- or OA-treated samples is represented as the normalized OA/DMSO index (the ratio of normalized spectral index of OA- and DMSO-treated samples).

### ANKRD13A is required for VCP recruitment during PINK1/Parkin-mediated mitophagy

ANKRD13A has been previously shown to interact with and recruit valosin-containing protein (VCP) in endolysosomal trafficking (20, 21). VCP is a member of the AAA-ATPase superfamily that functions to remodel protein complexes or lipid membranes *via* the extraction of its substrate proteins. It is involved in various cellular processes, including organellar homeostasis, protein quality control, trafficking, and chromatin regulation (27). VCP is crucial for PINK1/Parkin-mediated mitophagy and mitochondrial dynamics, and the mitochondrial translocation of VCP is essential for its mitophagy function (28). In addition, mutation of human *VCP* associated with degenerative disorders such as IBMPFD (Inclusion body myopathy with early onset Paget disease and frontotemporal dementia) (29), familial ALS (amyotrophic lateral sclerosis) (30), and Charcot-Marie-Tooth disease (31). All of these diseases are characterized by mitochondrial dysfunction (32, 33).

**Figure 5:**
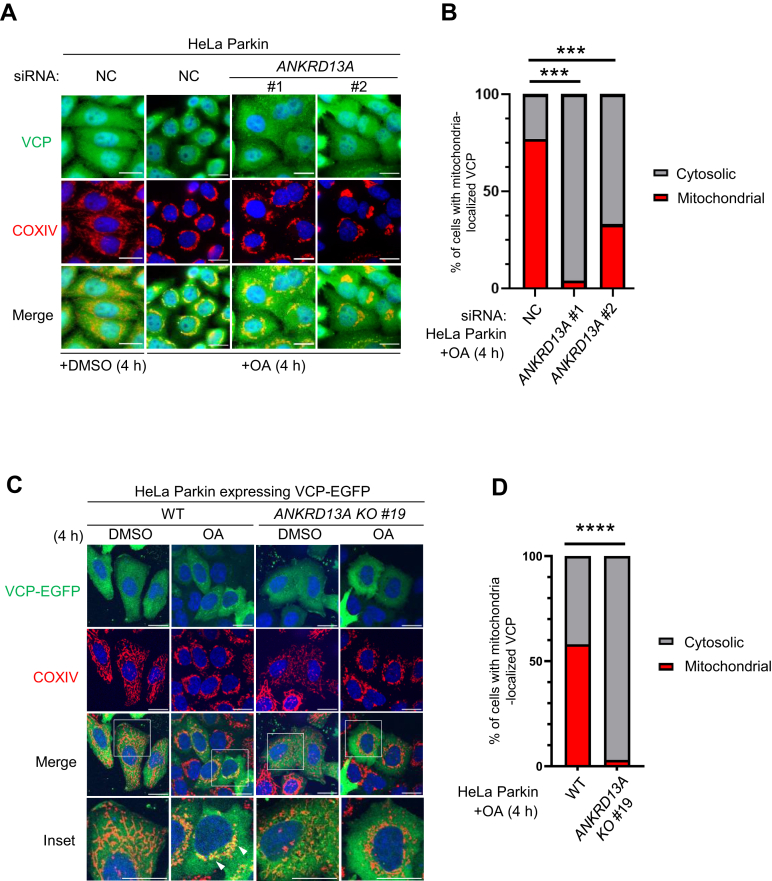
**ANKRD13A mediates mitochondrial recruitment of VCP during PINK1/Parkin-mediated mitophagy.***A*, representative images of mitochondrial recruitment of endogenous VCP upon mitophagy induction with OA. HeLa Parkin cells were treated with control (NC) or two different siRNA targeting *ANKRD13A*. Samples were incubated with either DMSO or OA for 4 h and immunofluorescently stained with anti-VCP or anti-COXIV (mitochondrial marker) antibodies. *B*, quantification of OA-induced mitochondrial recruitment of VCP in (*A*). At least 100 cells were analyzed per sample. Chi-square test, ∗∗∗*p* < 0.001. *C*, representative images of wild-type (WT) and *ANKRD13A* KO HeLa Parkin cells expressing VCP-EGFP. Cells were treated with either DMSO or OA for 4 h and immunofluorescently stained with anti-COXIV antibodies. *D*, quantification of OA-induced mitochondrial recruitment of VCP in (*C*). At least 120 cells were analyzed per sample. Chi-square test, ∗∗∗∗*p* < 0.0001. The scale bars represent 20 μm. ANKRD13A, ankyrin repeat domain-containing protein 13 A; DMSO, dimethyl sulfoxide; OA, oligomycin and antimycin; VCP, valosin-containing protein.

Given the known functions of ANKRD13A, we hypothesized that ANKRD13A facilitates PINK1/Parkin-mediated mitophagy by promoting the recruitment of VCP. The depletion of ANKRD13A *via* siRNA or CRISPR/Cas9-mediated KO resulted in reduced colocalization of endogenous VCP (Fig. 5, *A* and *B*) or exogenously expressed VCP (Figs. 5, *C*, *D* and S2*A*) with the mitochondria. The immunoprecipitation experiments showed that the interaction between ANKRD13A and VCP occurs under both basal and mitophagy-inducing conditions, and this interaction is not affected by mitophagy induction with OA (Fig. S2*B*). The interaction between ANKRD13A and VCP is UIM-dependent, consistent with a previous report (20). All these data suggested that ANKRD13A facilitates the recruitment of VCP to the depolarized mitochondria.

### Development of a biosensor to detect OMM rupture

The rupture of OMM is a critical step of PINK1/Parkin-mediated mitophagy. As previously mentioned, the ruptured OMM exposes IMM proteins for recognition by the autophagy system, promoting the clearance of the depolarized mitochondria (17, 18). However, the precise mechanism governing OMM rupture remains elusive. Previous research indicated that Parkin and proteasome are required for this process, suggesting that the removal of OMM protein through the action of Parkin and proteasome is a critical driving force behind membrane rupture. The removal of OMM proteins could destabilize the lipid bilayer, ultimately leading to membrane permeabilization and rupture. A key role of VCP during PINK1/Parkin-mediated mitophagy involves remodeling the OMM through the extraction of mitochondrial membrane protein for proteasome-dependent degradation (34, 35, 36). Having established that ANKRD13A acts as a mitophagy-regulated VCP recruitment factor, we were prompted to investigate the role of both ANKRD13A and VCP in the rupture of OMM.

**Figure 6:**
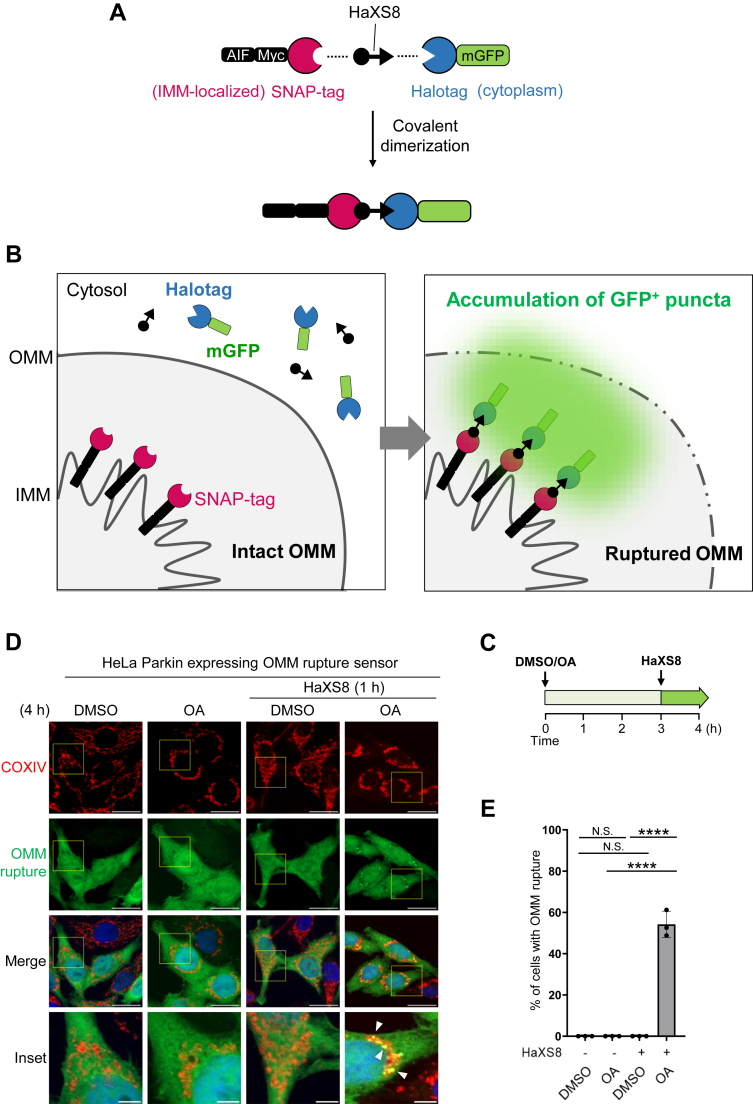

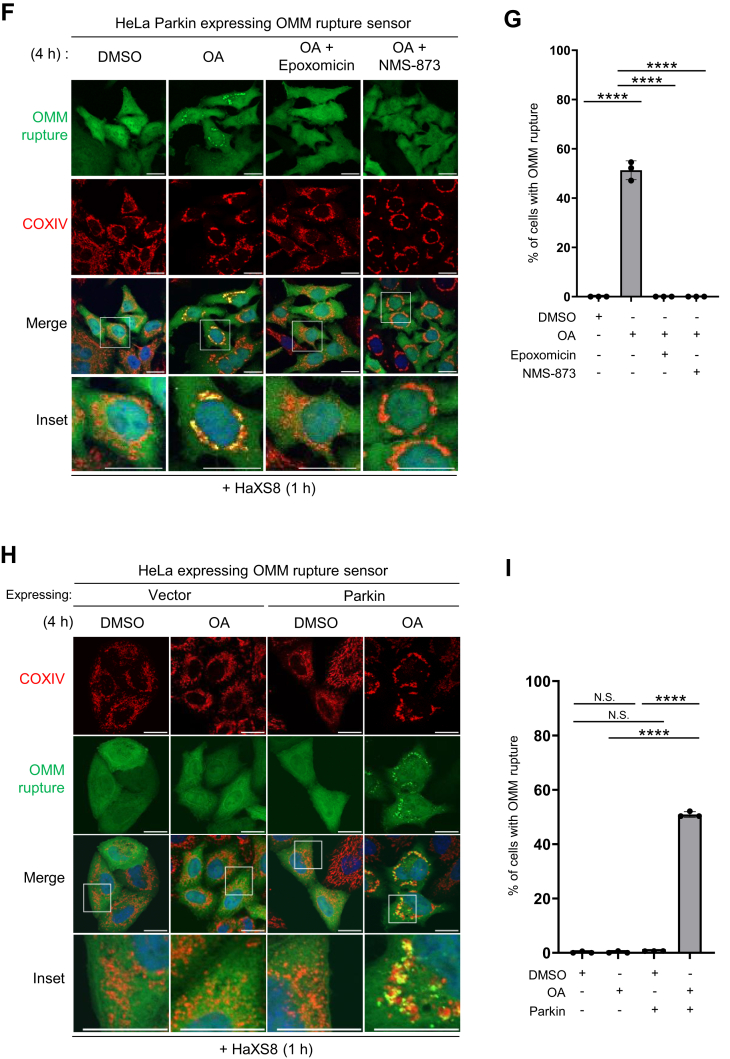
**Visualization of OMM rupture with a novel biosensor.***A*, scheme depicting the design of the OMM rupture sensor and HaXS8-mediated dimerization of AIF-Myc-SNAP-tag and Halotag-mGFP probes. *B*, the principle of the OMM rupture sensor. On the site where OMM becomes ruptured, cytosolic Halotag-mGFP gains access to the IMM and covalently conjugates with IMM-anchored AIF-SNAP-tag upon the presence of dimerizer HaXS8. The aggregated Halotag-mGFP on the OMM rupture site can be visualized as GFP-positive structures. *C*, for optimal detection of OMM rupture sites, cells were incubated with HaXS8 during the final hour of DMSO or OA treatment. *D*, representative images of HeLa Parkin cells expressing the OMM rupture sensor treated with either DMSO or OA for 4 h. HaXS8 (500 nM) was added to the cells 1 h prior to the analysis. At least 150 cells were analyzed per sample. The scale bars represent 20 μm. Inset scale bars represent 5 μm. *E*, quantification of OMM rupture shown in (*D*). Data presented as mean ± SD of a triplicate. N.S. non-significant, ∗∗∗∗*p* < 0.0001, One-way ANOVA with multiple comparisons. *F*, inhibition of proteasome and VCP activity blocks the OMM rupture. Representative images of HeLa Parkin cells expressing the OMM rupture sensor treated with DMSO/OA, epoxomicin (200 nM), or NMS-873 (10 μM) as indicated. HaXS8 (500 nM) was added to the cells 1 hour prior to the analysis. At least 150 cells were analyzed per sample. The scale bars represent 20 μm. *G*, quantification of OMM rupture shown in (*F*). Data presented as mean ± SD of a triplicate. ∗∗∗∗*p* < 0.0001, one-way ANOVA with multiple comparisons. *H*, Parkin is required for the formation of the OMM rupture puncta. Representative images showing HeLa cells expressing either empty vector (vector) or Parkin and the OMM rupture sensor. Cells were treated with either DMSO or OA for 4 h and incubated with HaXS8 (500 nM) during the last hour of DMSO/OA treatment. At least 150 cells were analyzed per sample. The scale bars represent 20 μm. *I*, quantification of OMM rupture shown in (*H*). Data presented as mean ± SD of a triplicate. N.S. non-significant, ∗∗∗∗*p* < 0.0001, One-way ANOVA with multiple comparisons. DMSO, dimethyl sulfoxide; IMM, mitochondrial inner membrane; mGFP, monomeric GFP; OA, oligomycin and antimycin; OMM, mitochondrial outer membrane.

Conventional methods to assess the integrity of the mitochondrial membranes include the protease protection assay and transmission electron microscopy (TEM). However, these methods are typically low throughput and labor-intensive. To streamline our investigation, we developed an assay that enables microscopic visualization of the OMM rupture sites *in* *cellulo* using a florescent protein probe that selectively marks the sites of IMM exposure. This biosensor utilizes chemical-induced dimerization (CID) of SNAP-tag and Halotag and consists of two probes: an IMM-targeted SNAP-tag (AIF-SNAP-tag) and a cytosolic Halotag fused to a monomeric GFP (Halotag-mGFP). Upon the rupture of OMM, Halotag-mGFP can gain access to the exposed AIF-SNAP-tag, enabling covalent conjugation of SNAP-tag and Halotag in the presence of the bifunctional dimerizer HaXS8 (37) (Fig. 6*A*). This reaction initiates the aggregation of Halotag-mGFP at the sites of OMM rupture, resulting in visible puncta on the mitochondria (Fig. 6*B*). To monitor OMM rupture during PINK1/Parkin-mediated mitophagy, we treated HeLa Parkin cells expressing the OMM rupture biosensor with either DMSO or OA for a total of 4 h. During the final hour of DMSO/OA treatment, HaXS8 was added to initiate the labeling of OMM rupture sites (Fig. 6*C*). Our results showed that treatment with OA and HaXS8 led to a significant formation of mGFP-positive puncta on the damaged, perinuclear clustered mitochondria (Fig. 6, *D* and *E*). In contrast, treatment with either OA or HaXS8 alone did not yield mGFP-positive puncta, indicating that the labeling of the OMM rupture site requires both mitophagy induction with OA and HaXS8-dependent dimerization.

Using this new biosensor, we reevaluated the dependency of OMM rupture on proteasome and Parkin. As anticipated, the inhibition of proteasome with epoxomicin (Fig. 6, *F* and *G*) or the absence of Parkin (Fig. 6, *H* and *I*) prevented the formation of mGFP-positive puncta upon OA treatment. Consistent with our existing understanding of OA-induced OMM rupture, the signals of OMM rupture predominantly colocalized with Parkin-positive mitochondria (Fig. S3*A*). In addition, a subset of OMM rupture signals overlapped with LC3B- and OPTN-positive mitochondria, indicating the OMM-ruptured mitochondria are subjected to autophagic removal (Fig. S3, *B* and *C*). Taken together, our innovative fluorescent OMM rupture biosensor effectively marks the ruptured mitochondria, providing an excellent tool for investigating the regulation of OMM rupture during PINK1/Parkin-mediated mitophagy.

### ANKRD13A and other VCP recruitment factors are required for OA-induced OMM rupture

**Figure 7:**
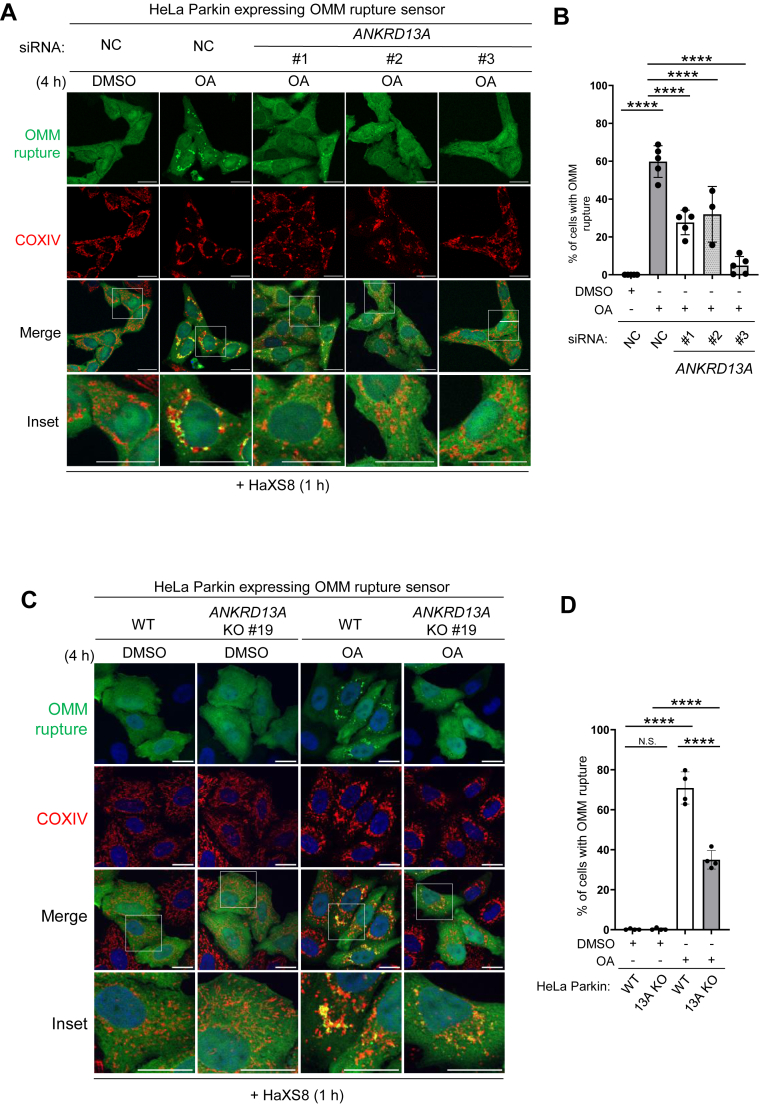

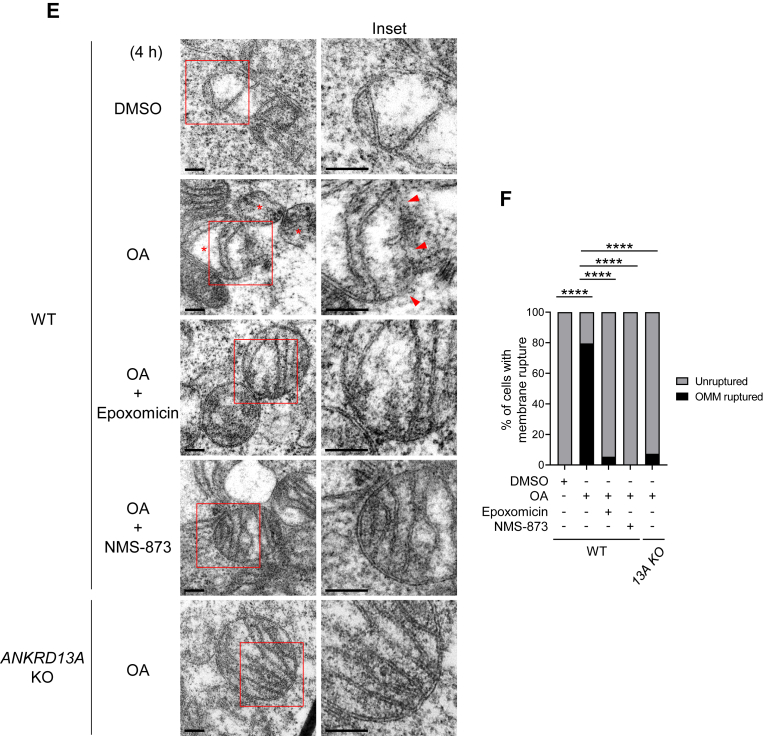

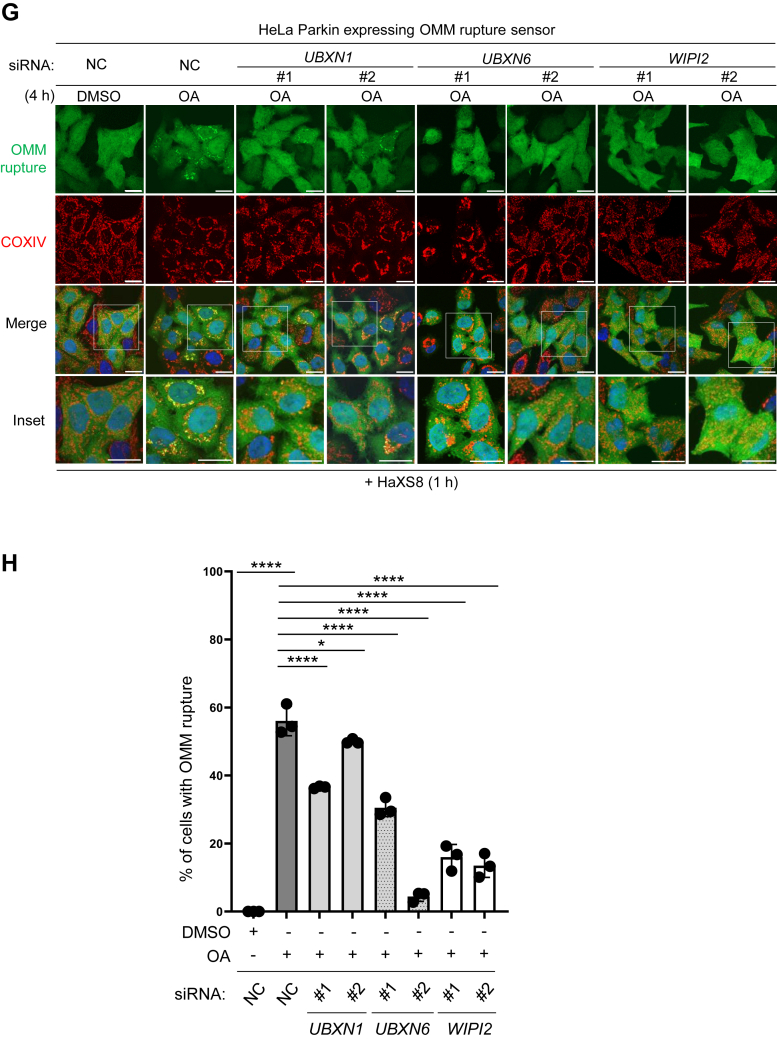
**ANKRD13A and other VCP recruitment factors are required for the OMM rupture during PINK1/Parkin-mediated mitophagy.***A*, representative images of HeLa Parkin cells expressing the OMM rupture sensor. Cells were treated with control (NC) or three different *ANKRD13A* siRNA and incubated with DMSO/OA for 4 h, with HaXS8 (500 nM) added during the last hour of DMSO/OA treatment. At least 150 cells were analyzed per sample. The scale bars represent 20 μm. *B*, quantification of OMM rupture in the experiment shown in (*A*). Data presented as mean ± SD of a triplicate. ∗∗∗∗*p* < 0.0001, One-way ANOVA with multiple comparisons. *C*, representative images of wild-type or *ANKRD13A* KO HeLa Parkin cells expressing the OMM rupture sensor treated with DMSO/OA and HaXS8 as in (*A*). At least 150 cells were analyzed per sample. The scale bars represent 20 μm. *D*, quantification of OMM rupture in the experiment shown in (*C*). Data presented as mean ± SD of a triplicate. N.S. non-significant, ∗∗∗∗*p* < 0.0001, One-way ANOVA with multiple comparisons. *E*, representative TEM micrographs showing the mitochondria in wild-type (WT) or *ANKRD13A* KO (13A KO) cells treated with DMSO, OA, or OA + epoxomicin (400 nM), OA + NMS-873 (10 μM) as indicated for 4 h. *Asterisks* denote the ruptured mitochondria. *Arrows* mark the site of rupture. The scales bars represent 200 nm. *F*, quantitation of mitochondrial membrane rupture in (*E*). At least 40 mitochondria were analyzed per sample. ∗∗∗∗*p* < 0.0001, Chi-square test. *G*, representative images of HeLa Parkin cells expressing the OMM rupture sensor treated with control (NC) or siRNA targeting *UBXN1*, *UBXN6*, or *WIPI2*. Cells were incubated with DMSO/OA and HaXS8 as in (*A*) and analyzed. At least 200 cells were analyzed per sample. The scale bars represent 20 μm. *H*, quantification of OMM rupture in (*G*). Data presented as mean ± SD of a triplicate. ∗*p* < 0.05, ∗∗∗∗*p* < 0.0001, One-way ANOVA with multiple comparisons. ANKRD13A, ankyrin repeat domain-containing protein 13 A; DMSO, dimethyl sulfoxide; OA, oligomycin and antimycin; OMM, mitochondrial outer membrane; TEM, transmission electron microscopy.

With our new OMM rupture sensor, we explored whether ANKRD13A and VCP are involved in the OMM rupture. Remarkably, the inhibition of VCP with NMS-873 (an allosteric VCP inhibitor) nearly completely halted the formation of OMM rupture puncta (Fig. 6, *F* and *G*), suggesting that VCP is an absolute requirement for the OMM rupture. The depletion of ANKRD13A with three different siRNAs (Fig. 7, *A* and *B*) or the knockout of ANKRD13A (Fig. 7, *C* and *D*) significantly reduced the frequency of the OMM rupture, suggesting that ANKRD13A is also required for OMM rupture.

To further verify this observation, we employed TEM to examine the ultrastructure of mitochondria in HeLa Parkin cells undergoing OA-induced mitophagy. Following OA treatment, the majority of mitochondria showed ruptured OMM, but proteasome inhibition with epoxomicin or VCP inhibition with NMS-873 nearly completely prevented this occurrence. Likewise, the membrane rupture is almost entirely abrogated in *ANKRD13A* KO cells (Fig. 7, *E* and *F*), further confirming the essential role of ANKRD13A in OMM rupture. These direct ultrastructural observations corroborate and strengthen our findings obtained using the OMM rupture biosensor, thereby validating its utility and specificity in capturing mitochondrial membrane rupture events.

Previous studies have identified UBXN1, UBXN6, and WIPI2 as VCP recruitment factors that translocate to the depolarized mitochondria, promoting PINK1/Parkin-mediated mitophagy and OMM protein degradation (34, 35, 36), indicating that these factors may also contribute to the OMM rupture. Indeed, the depletion of UBXN1, UBXN6, and WIPI2 with siRNA (Fig. S4, *A*–*C*) decreased the frequency of OMM rupture (Fig. 7, *G* and *H*). Notably, the depletion of WIPI2 had a pronounced inhibitory effect on the OMM rupture; the level of inhibition observed was comparable to that caused by VCP inhibition (Fig. 6*G*). In contrast, UBXN1 and UBXN6 depletion only resulted in a mild reduction in OMM rupture. Given the efficiencies among *UBXN1* and *UBXN6* siRNAs, it is difficult to ascertain whether these two factors are as critical as WIPI2. Nevertheless, it is clear that ANKRD13A, as well as other mitophagy-regulated VCP recruitment factors, is required for the rupture of the OMM during PINK1/Parkin-mediated mitophagy.

## Discussion

In this study, we identified a novel function of ANKRD13A that goes beyond its original role in endolysosomal trafficking. During PINK1/Parkin-mediated mitophagy, ANKRD13A relocalizes to the depolarized mitochondria to mediate the recruitment of VCP, an essential protein for mitochondrial quality control. Mechanistically, the UIMs of ANKRD13A are essential for its binding to VCP (Fig. S2*B*) as well as its interaction with mitochondrial proteins PHB2, ATAD3A, CHCHD3, IMMT, and PGAM5. (Figure 3, Figure 4*D* and 4, *E*, *F*). Functionally, the UIMs are critical for OA-induced mitophagy (Fig. 3, *E*–*H*), suggesting that ANKRD13A promotes mitophagy *via* UIM-dependent interactions with these ubiquitylated mitochondrial proteins and VCP. Previous studies have demonstrated that the four tandem UIMs located in the C terminus of ANKRD13A specifically recognize K63-linked ubiquitin chains (21). It is well-established that UIM domains generally display low affinity to ubiquitin in isolation and lack intrinsic linkage specificity. In contrast, tandem UIM arrangements with appropriate spacing and linker sequences significantly enhance affinity through multivalent binding and provide linkage specificity (38, 39). Based on these observations, we propose that each UIM contributes partially and cooperatively to the recruitment of ANKRD13A during PINK1/Parkin-mediated mitophagy. Although the deletion of the AR did not impact the mitochondrial recruitment of ANKRD13A (Fig. 3, *B* and *C*), it partially reduced the interaction between ANKRD13A and VCP (Fig. S2*B*) and impaired OA-induced mitophagy (Fig. 3, *E*–*H*). Thus, it appears that the AR primarily mediates the interaction with VCP but is not involved in the recruitment of ANKRD13A to the depolarized mitochondria.

ANKRD13A shares functional similarities with the mitophagy receptors OPTN and NDP52, which play a major role in the PINK1/Parkin-dependent pathway. These proteins preferentially recognize polyubiquitin chains, are recruited to depolarized mitochondria, and participate in vesicle trafficking (21, 40). The convergence of their roles in mitophagy suggests a potential interrelationship. Given the role of ANKRD13A in endolysosomal trafficking (20), it is unknown whether ANKRD13A facilitates the trafficking of OPTN- or NDP52-containing vesicles to mitochondria. Furthermore, as a VCP recruitment factor, ANKRD13A may facilitate the VCP/proteasome-dependent removal of K48-linked ubiquitin chains, which could result in an enrichment of K63-linked ubiquitin chains, promoting the mitochondrial recruitment of OPTN and NDP52. Thus, we examined the OA-induced mitochondrial relocalization of epitope-tagged OPTN and NDP52 in wild-type and *ANKRD13A* KO cells. Our findings indicated that the loss of ANKRD13A did not hinder the mitochondrial translocation of either OPTN or NDP52 upon OA-induced mitophagy (Fig. S6), suggesting that OPTN/NDP52 are unlikely to be regulated by ANKRD13A.

The ANKRD13A-interacting mitochondrial proteins (PHB2, ATAD3A, CHCHD3, IMMT, and PGAM5) have different localizations. Specifically, PHB2, IMMT, and CHCHD3 are IMM proteins, while ATAD3A is a transmembrane protein that spans both the OMM and IMM. PGAM5 has been shown to associate with both OMM and IMM. Since ANKRD13A is required for OMM rupture, we proposed that during OA-induced mitophagy, ANKRD13A may initially associate with its OMM interacting proteins, including ATAD3A, PGAM5, or other unidentified proteins, and facilitate VCP-dependent permeabilization. Once the IMM is exposed, ANKRD13A can then interact with its binding partners on the IMM, such as PHB2, IMMT, CHCHD3, and PGAM5, to further enhance VCP recruitment and the rupture of the OMM.

Moreover, PHB2, IMMT, ATAD3, and CHCHD3 are IMM scaffold proteins essential for maintaining the structure and function of the IMM. Prohibitins and ATAD3 are critical for the maintenance of cristae structure and ensure the proper formation of respiratory chain complexes (41, 42). IMMT and CHCHD3 are key subunits of a protein complex known as MICOS (mitochondrial contact site and cristae organizing system), which is vital for cristae morphogenesis and tethering of the IMM and OMM (43, 44). Thus, targeting ANKRD13A to these IMM proteins may also facilitate VCP-dependent disassembly of IMM scaffold protein complexes, promoting mitochondrial disintegration during the late phase of PINK1/Parkin-mediated mitophagy.

VCP plays a crucial role in PINK1/Parkin-mediated mitophagy and mitochondrial quality control (28). However, the precise mechanisms by which VCP facilitates mitophagy are not yet fully understood. First, previous studies have suggested that VCP can remove specific ubiquitylated OMM proteins, including MFN1 and MFN2, to promote mitochondrial fission, a process essential for mitophagy (28). Second, it has been proposed that during selective autophagy, VCP mediates proteasome-dependent removal of K48-linked ubiquitin chains. This removal leads to an enrichment of K63-linked ubiquitin chains, which helps recruit ubiquitin-binding mitophagy receptors that bind to these K63-linked chains. Third, VCP also promotes autophagosome biogenesis around the substrates of selective autophagy (27). In this study, we first demonstrated that the promotion of OMM rupture is also a VCP-dependent process. The pharmacological inhibition of proteasome or VCP completely prevented the formation of OMM rupture signals (Fig. 6, *F* and *G*), indicating that VCP-mediated, proteasome-dependent degradation is critical for the OMM rupture. Consistent with this, the depletion of mitophagy-regulated VCP recruitment factors (ANKRD13A, UBXN1, UBXN6, and WIPI2) inhibits OMM rupture (Fig. 7). Previous studies proposed that upon mitochondrial depolarization, increased intramitochondrial and IMM pressure, along with proteasome-dependent removal of OMM protein, contribute to membrane destabilization and the eventual rupture (45). As VCP has numerous mitochondrial substrates, we do not know the removal of which specific VCP substrate(s) is responsible for the OMM rupture. With our novel OMM rupture sensor, it will be possible to conduct genetic screens on VCP substrates to identify the mechanisms underlying OMM permeabilization during PINK1/Parkin-mediated mitophagy.

To better present the unique role of WIPI2 among core autophagy proteins in OMM rupture, we conducted genetic and pharmacological inhibition of autophagy genes along with WIPI2 and examined the occurrence of the OMM rupture. Surprisingly, the depletion of core autophagy proteins Beclin 1 (Fig. S5, *A* and *B*) and ATG5 (Fig. S5, *C* and *D*), as well as the pharmacological inhibition of VPS34 (catalytic subunit of class III phosphatidylinositol-3-kinase) (Fig. S5, *E* and *F*), led to a modest decrease in the OMM rupture signal. One might anticipate that impaired autophagy would result in increased OMM rupture signals, as the damaged mitochondria would not be efficiently removed. However, our results suggested otherwise. These findings indicate that canonical autophagy factors contribute to the OMM rupture. Still, WIPI2 exerts a more pronounced effect, which is consistent with its unique function in recruiting VCP to depolarized mitochondria. The partial involvement of general autophagy machinery suggests that OMM rupture is likely driven by both WIPI2-dependent VCP recruitment and autophagic processes. Further investigation is warranted to delineate the mechanistic interplay.

A recent whole-genome sequencing study revealed that ANKRD13D, a member of the ANKRD13 family that shared high sequence homology and overlapping functions with ANKRD13A (20, 21), is associated with the risk and progression of Alzheimer’s disease (AD) (46). The study identified seven deleterious *ANKRD13D* mutations that were predicted to be missense mutations potentially affecting its ubiquitin-binding abilities. *ANKRD13A* was also considered a potential candidate gene associated with AD in the same study, although it did not achieve statistical significance in later analyses. These findings indicated that impaired mitophagy functions of ANKRD13 family proteins may contribute to the pathogenesis of AD.

Here, we developed a HaXS8-based CID fluorescent biosensor to microscopically visualize the site of OMM rupture. Compared with other CID systems that we evaluated during assay development, this assay provided a more consistent readout and demonstrated superior capability in capturing OMM rupture events. This is likely due to the nature of HaXS8 as an irreversible dimerizer that allows for the accumulation of OMM rupture signals during labeling, thereby improving detection sensitivity. However, while the irreversibility of HaXS8-mediated dimerization boosts detection capabilities, it also limits the applicability of this method for time-course analysis of OMM rupture. We observed that adding HaXS8 prior to OMM rupture typically resulted in diminished OMM rupture signals. We postulated that in situations where the OMM has not yet ruptured, the IMM-localized SNAP-tag and cytosolic Halotag remain separated. The reaction with HaXS8 results in HaXS8-labeled SNAP-tag and Halotag that can no longer conjugate with one another, resulting in a “saturation” of the probes, which negatively impacts detection. As such, this reporter is better suited for end point analysis rather than continuous monitoring of OMM rupture. In addition, because this method relies on the formation of mGFP-positive puncta at the rupture sites, minor OMM ruptures may not accumulate enough mGFP probes to form visible puncta. Thus, future improvements of this biosensor are warranted.

With the new technique, we are the first to acquire temporal and spatial information regarding the OMM rupture during PINK1/Parkin-mediated mitophagy. We observed that the OMM rupture signals appear 2 h after the OA treatment, a time frame that follows the initial PINK1/Parkin activation and OMM ubiquitylation (47). During PINK1/Parkin-mediated mitophagy, depolarized mitochondria form aggregates in perinuclear regions following damage-induced mitochondrial fission. This aggregation is dependent on PINK1/Parkin-mediated ubiquitylation of mitochondria and ubiquitin-binding autophagy adaptor p62/SQSTM1 (48, 49). We found that the OMM rupture signals appear almost exclusively on the perinuclear-clustered mitochondria; however, not all the perinuclear-clustered mitochondria showed signs of OMM rupture. This observation suggests that OMM rupture likely occurs either after or concurrently with the compaction of depolarized mitochondria to the perinuclear region. However, due to the limitation of this new technique, we were unable to pinpoint the exact timing of OMM rupture in relation to other molecular events of PINK1/Parkin-mediated mitophagy by using time-lapse live-cell imaging.

Membrane rupture commonly serves as a trigger for various forms of selective autophagy, including the autophagy of the damaged lysosome (lysophagy) and intracellular pathogens that undergo endosomal escape (xenophagy). The site of endomembrane rupture acts as a danger signal to be recognized by cellular machinery responsible for organellar repair, ubiquitylation, proteasomal, or autophagic clearance (50, 51). Why do cells bother to rupture the OMM for PINK1/Parkin-mediated mitophagy when the ubiquitylation of OMM protein and the recruitment of autophagy receptors should suffice to recruit the autophagic membrane to the depolarized mitochondria? One possibility is that the rupture of OMM releases additional signals to support mitophagy, ensuring proper disposal of dysfunctional mitochondria. Moreover, OMM ubiquitylation can be reversed by deubiquitinating enzymes to attenuate mitophagy. In this sense, the rupture of OMM may represent a “point of no return” for mitophagy. Future studies focused on understanding the mechanisms and molecular events associated with OMM rupture will provide new insights into its role in PINK1/Parkin-mediated mitophagy.

## Experimental procedures

### Expression plasmid and cloning

PHB2-StrepII-FLAG expressing construct was generated by PCR amplification of human *PHB2* and subcloned to pLenti-IRES-Puro. 3xFLAG-ANKRD13A-expressing construct was generated by cloning human ANKRD13A complementary DNA (cDNA) to pBICEP-CMV, followed by a subcloning of 3xFLAG-ANKRD13A to pLenti-IRES-Neo vector. Constructs expressing ΔAR (102–590 a.a.), ΔUIM (1–481 a.a.), or mUIM (A490G/S494A/A526G/S530A/A556G/S560A/V581G/S585A) mutants were generated by Gibson assembly. Flag-tagged VCP construct was made by subcloning VCP to pLenti-IRES-Puro. For the OMM rupture sensor, fragments containing AIF (1–90 a.a.) were amplified from pcDNA3 AIF 1 to 90 mCherry (a gift from Stephen Tait, Addgene #67530) and Myc-SNAP^f^-T2A-mGFP-Halotag7 were gene synthesized. Fragments were Gibson assembled with pLenti-IRES-Puro.

### Mammalian cells

HeLa and SH-SY5Y cells were originally obtained from the American Type Culture Collection. HeLa Parkin cells were generated by stable transfection of pIRES-hyg3 vector (Clontech) with human Parkin cDNA. HeLa Parkin cells expressing wild-type or mutant 3xFLAG-ANKRD13A were generated by lentiviral transduction of pLenti-IRES-Neo vector carrying wild-type or mutant ANKRD13A cDNA into HeLa Parkin cells. HeLa Parkin PHB2-StrepII-FLAG cells were generated by lentiviral transduction of pLenti-PHB2-StrepII-FLAG-IRES-Puro into HeLa Parkin cells.

### Chemicals

The working concentrations of the chemicals are as follows: oligomycin (2.5 μM) together with antimycin (250 nM) (OA), DFP (1 mM), epoxomicin (200 or 400 nM), N-Ethylmaleimide (NEM, 10 mM), HaXS8 (500 nM), NMS-873 (10 μM), and PIK-III (5 μM). DFP was dissolved in distilled water, and all other chemicals were dissolved in DMSO. All stock solutions were stored at −80 °C in small aliquots.

### Antibodies

For immunofluorescence analysis, primary antibodies were mouse rabbit anti-COXIV (Proteintech 11242-1-AP, 1:1000), rabbit anti-COX IV (Abcam ab16056, 1:1000), mouse anti-ATP5B (Millipore MAB3494, 1:1000), mouse anti-FLAG M2 (Sigma-Aldrich F1804, 1:1000), rabbit anti-HA tag antibody (Cell Signaling Technology, 3724S, 1:1000), mouse anti-Parkin (Cell signaling Technology, 4211, 1:1000) and mouse anti-V5 tag (Invitrogen R96025, 1:250), and mouse anti-VCP (Santa Cruz sc-57492, 1:1000). The secondary antibodies were Donkey anti-rabbit IgG AlexaFluor 594 (Invitrogen A21207, 1:750), Donkey anti-mouse IgG Alexa Fluor 488 (Invitrogen A21202, 1:750), goat anti-mouse IgG1 AlexaFluor 488 (Invitrogen A21121, 1:750), and goat anti-mouse IgG2b AlexaFluor 488 (Invitrogen A21145, 1:750).

For Western blot analysis, primary antibodies were rabbit anti-ANKRD13A (Sigma-Aldrich, HPA039488, 1:1000), rabbit anti-ATAD3A/B (Proteintech 16610-1-AP, 1:1000), rabbit anti-ATG5 (Cell signaling 12994, 1:1000), rabbit anti-ATG7 (Sigma-Aldrich, A2856, 1:1000), mouse anti-ATP5B (Millipore, MAB3494, 1:1000), mouse anti-β-actin (Santa Cruz sc-47778, 1:5000), rabbit anti-CHCHD3 (Proteintech 25625-1-AP, 1:1000), rabbit anti-COX IV (Abcam ab16056, 1:10,000), mouse anti-Flag M2 (Sigma-Aldrich F1804, 1:2000), mouse anti-His (Santa Cruz sc-8036, 1:1000), mouse anti-HSP60 (Proteintech 66041-1-Ig, 1:10,000), rabbit anti-IMMT (Proteintech, 10179-1-AP, 1:5000), rabbit anti-LDHA (Proteintech 19987-1-AP, 1:10,000), mouse anti-PHB2 (Santa Cruz, sc-133094, 1:10,000), rabbit anti-PHB2 (Proteintech, 12295-1-AP, 1:10,000), mouse anti-TOMM20 (Santa Cruz, sc-17764, 1:1000), mouse anti-VCP (Santa Cruz, sc-57492, 1:1000), 1:1000), and rabbit anti-PGAM5 (Proteintech, 28445-1-AP, 1:2000). The secondary antibodies were IRDye 800CW goat anti-mouse IgG (LI-COR, 926-32210, 1:7500), IRDye 680RD goat anti-mouse IgG (LI-COR, 926-68071, 1:7500), Peroxidase AffiniPure goat anti-mouse IgG (H + L) (Jackson immune Research, 115-035-003, 1:20,000), and Peroxidase AffiniPure goat anti-rabbit IgG (H + L) (Jackson immune Research, 111-035-003, 1:10,000). For the detection of endogenous ANKRD13A and PHB2 in immunoprecipitant, as well as the immunopurification of mitochondria, EasyBlot anti-rabbit IgG (horseradish peroxidase, HRP) (GeneTex GTX221666-01, 1:5000) and FlexAble HRP Antibody Labeling kit (Proteintech, KFA005) were used.

### Lentivirus preparation

pLenti-PHB2-StrepII-FLAG-IRES-Puro and pLenti-3xFLAG-ANKRD13A-IRES-Neo with the AR or UIM deletion/mutations were cotransfected with helper plasmids pCMVΔR8.91 and pMD.G (RNAi Core, Academia Sinica) into HEK293FT cells with T-Pro Non-liposome Transfection Reagent II (T-Pro Biotechnology). Forty-eight hours post transfection, lentiviral supernatant was filtered through a 0.45 μm filter and infected the target cells in the presence of polybrene at 8 μg/ml. Twenty-four hours later, the virus-containing medium was removed and replaced with fresh medium containing 0.5 μg/ml puromycin and/or 500 μg/ml G418 and selected for at least 2 weeks.

### Tandem affinity purification of PHB2-containing protein complex

In addition, 10^8^ HeLa Parkin PHB2-StrepII-FLAG cells were treated with either DMSO or OA for 4 h. The cells were harvested in cell lysis buffer [25 mM Hepes (pH 7.4), 150 mM NaCl, 1 mM EDTA] supplemented with 1× cOMPLETE protease inhibitor cocktail (Roche # 04693159001), and 1× HALT phosphatase inhibitor cocktail (Thermo Fisher Scientific, #78440). The PHB2-containing protein complex was first purified with Strep-Tactin Superflow resin (IBA Lifesciences, #2-1208-025) and followed by a second purification with anti-FLAG M2 affinity gel (Sigma-Aldrich A2220).

### Isolation of ANKRD13A-containing protein complex

Subsequently, 2 × 10^8^ HeLa Parkin cells were treated with either DMSO or OA 4 h, and cell lysates were prepared with cell lysis buffer supplemented with 1× cOMPLETE protease inhibitor cocktail, phosphatase inhibitors, and 10 mM NEM. Cell lysates (60 mg) were incubated with 100 μl bed volume of prewashed anti-FLAG M2 affinity gel at 4 °C overnight. The beads were washed 5 min five times with cell lysis buffer supplemented with 0.1 mg/ml AEBSF, phosphatase inhibitor, and 10 mM NEM, and washed once with 50 mM NaCl. The proteins were eluted by 0.5 N NH_4_OH/0.5 mM EDTA solution and freeze-dried.

### RNAi-mediated knockdown

All siRNA-mediated knockdowns were performed at a final concentration of 10 nM DsiRNA using Lipofectamine RNAiMAX (Invitrogen #13778150) according to the manufacturer’s instructions. After 48 h, gene knockdown efficiency was assessed by Western blot analysis. Dicer substrate siRNAs (DsiRNA) were purchased from IDT DNA. The siRNA sequences are as follows:

NC1 (Nontargeting control), 5′-CGUUAAUCGCGUAUAAUACGCGUAT-3′; 3′- CAGCAAUUAGCGCAUAUUAUGCGCAUA-5′;

*ANKRD13A* #1 (hs.Ri.ANKRD13A.13.2), 5′- ACUCUGGACUUGAUGAAGCCAAAAA-3′; 3′- AGUGAGACCUGAACUACUUCGGUUUUU-5′;

*ANKRD13A* #2 (hs.Ri.ANKRD13A.13.3), 5′-GAUGAAGAUUACGAGAUAAUGCAGT-3′; 3′-UUCUACUUCUAAUGCUCUAUUACGUCA-5′;

*ANKRD13A* #3 (hs.Ri.ANKRD13A.13.4), 5′-GCACUGAAAUAACCUGGUAAACAAC-3′; 3′- GACGUGACUUUAUUGGACCAUUUGUUG-5′;

*ATG7* (hs.DsiATG7-1), 5′-GGGUUAUUACUACAAUGGUGACUCT-3′; 3′-UUCCCAAUAAUGAUGUUACCACUGAGA-5′.

*BECN1 #1* (hs.Ri.BECN1.13.1), 5′-AGUACAUGUUUACAAUACCAAAAAA-3′; 3′-UUUUUUGGUAUUGUAAACAUGUACUGU-5′.

*BECN1 #2* (hs.Ri.BECN1.13.2), 5′-GAAGAAAACCAACGUCUUUAAUGCA-3′; 3′-UGCAUUAAAGACGUUGGUUUUCUUCAG-5′.

PINK1 (hs.Ri.PINK1.13.1), 5′-AGUCACUUACAGAAAAUCCAAGAGA-3′; 3′-UCUCUUGGAUUUUCUGUAAGUGACUGC-5′.

*UBXN1* #1 (hs.Ri.UBXN1.13.1), 5′-ACCCCUUCAUCUUUGAUAAAGCACT-3′; 3′-ACUGGGGAAGUAGAAACUAUUUCGUGA-5′.

*UBXN1* #2 (hs.Ri.UBXN1.13.3), 5′-GACGGCUCUUGAGAGUCUCAUCGAG-3′; 3′-GACUCCGAGAACUCUCAGAGUAGCUC-5′.

*UBXN6* #1 (hs.Ri.UBXN6.13.1), 5′-CAUAGACCUGCAGUUAGUAAAUCAT-3′; 3′-GUGUAUCUGGACGUCAAUCAUUUAGUA-5′.

*UBXN6* #2 (hs.Ri.UBXN6.13.2), 5′-GGAGAAGUACCGGAAGAUCAAGCTG-3′; 3′-CUCCUCUUCAUGGCCUUCUAGUUCGAC-5′.

*WIPI2* #1 (hs.Ri.WIPI2.13.1), 5′-GAGGUUCUUUCUGAUACUAAAAACC-3′; 3′-CGCUCCAAGAAAGACUAUGAUUUUUGG-5′.

*WIPI2* #2 (hs.Ri.WIPI2.13.2), 5′-GUCUGGAAACGACCAAUGAGAUCTT-3′; 3′-GUCAGACCUUUGCUGGUUACUCUAGAA-5′.

For shRNA-mediated knockdown, Dulbecco's modified Eagle's medium containing virus supernatant at a multiplicity of infection of three, and 10 μg/ml polybrene was used to infect target cells. The virus-containing medium was replaced with fresh media 24 h after infection. Forty-eight hours after infection, the infected cells were selected with puromycin at 0.5 μg/ml (HeLa) or 1.0 μg/ml (SH-SY5Y). shRNA lentiviruses were obtained from the RNA Technology Platform and Gene Manipulation Core. The shRNA target sequences are as follows:

Control (TRC2.void), 5′-AGTTCAGTTACGATATCATGTCTCGAGACATTCGCGAGTAACTGAACTTTTTTG-3′

ATG5 (TRCN0000151963), 5′-CCTGAACAGAATCATCCTTAA-3′.

ANKRD13A#1 (TRCN0000167374), 5′-GATATCACATTGCTGGGATTT-3′.

ANKRD13A#2 (TRCN0000431140), 5′-GAGCATCATCACTTTCCATTA-3′.

### Generation of CRISPR/Cas9 KO cell lines

For the KO of *ANKRD13A*, we used two guide RNA sequences targeting the exons I and III of *ANKRD13A*: 5′-TAGTCTGGAAAAACGACTAC-3′ and 5′-TGTTGTGGTAGTCTCGATGT-3′. The synthetic oligonucleotides were annealed and cloned into BbsI-digested pX459. HeLa Parkin cells were transfected with the pX459 carrying ANKRD13A guide RNA and followed by a 4-day puromycin selection at 0.5 μg/ml. The cells were recovered in an antibiotic-free medium and serially diluted to allow the growth of single colonies. The gene KO was confirmed by Western blot and validated with PCR followed by Sanger sequencing.

### Co-immunoprecipitation

For co-immunoprecipitation with FLAG-tagged proteins, cells expressing FLAG-tag expressing constructs were treated with either DMSO or OA (2.5 μM oligomycin + 250 nM antimycin A) for 4 h. Cells were collected in cell lysis buffer [25 mM Hepes (pH 7.4), 150 mM NaCl, and 1 mM EDTA] supplemented with 1× cOMPLETE protease inhibitor cocktail (Roche # 04693159001), a mixture of phosphatase inhibitor (50 mM sodium fluoride, 1 mM β-glycerophosphate, 1 mM sodium pyrophosphate, and 1 mM sodium orthovanadate), and 10 mM NEM. Samples were incubated on ice for 20 min and centrifuged at 16,000*g* for 20 min. The supernatants were diluted with an appropriate volume of cell lysis buffer, incubated with anti-FLAG M2 antibody (F1804, Sigma-Aldrich) at 1:1000 dilution and Protein G Mag Sepharose Xtra (Cytiva #28967070) preblocked with 5% bovine serum albumin in TBST. Samples were incubated overnight at 4 °C. Beads were washed three times with cell lysis buffer supplemented with AEBSF and analyzed by Western blot analysis. For the co-immunoprecipitation of endogenous PHB2 and ANKRD13A, cells were incubated with 2 mM DSP [dithiobis(succinimidyl propionate)] in D-PBS at 4 °C for 2 h prior to cell lysis.

### Western blot analysis

Cell lysates were prepared in 1× Laemmli buffer containing 2.5% β-mercaptoethanol and boiled at 95 °C for 5 min. The samples were separated on SDS-PAGE and transferred to either polyvinylidene fluoride or nitrocellulose membrane. Membranes were blocked with 5% bovine serum albumin in TBST (with 0.1% Tween-20) and incubated with primary and either HRP- or IRDye800CW/680RD-conjugated antibodies. Signals were visualized by either the LAS-4000 image system (GE Healthcare) or the Odyssey Imaging System (LI-COR).

### Immunopurification of mitochondria

Cells were washed with ice-cold D-PBS and collected in Buffer A (0.25 M sucrose, 10 mM Tris–HCl (pH 7.5), 10 mM KCl, 1.5 mM MgCl_2_, and 1 mM EDTA) supplemented with 1× cOMPLETE protease inhibitor cocktail and a mixture of phosphatase inhibitors. The samples were incubated on ice for 15 min and homogenized in either a 1 ml syringe with 28G needles or a Dounce homogenizer with pestle B with 25 strokes. The cell homogenates were centrifuged at 700*g* for 5 min at 4 °C to remove nuclei and unbroken cells, and the resulting PNS were collected for immunopurification. PNS (1000 μg) were incubated with anti-TOMM20 antibodies (Santa Cruz scbt-17764, 1:200 and Proteintech 80501-1-RR, 1:400) at 4 ˚C for 1 h and followed by an additional 1 h incubation with 30 μl Protein G Dynabeads (Thermo Fisher Scientific 10003D). Immunoprecipitants were finally washed 5 min for three times with Buffer A and boiled in 1× Laemmli buffer for subsequent analysis.

### Mitochondrial fractionation

Mitochondrial fractions were isolated using differential centrifugation with Qproteome mitochondrial isolation kit (Qiagen #37612) according to the manufacturer's instructions. The isolated fractions were resuspended in 1× Laemmli buffer for Western blot analysis.

### Light microscopy

For immunofluorescence analysis of cultured cells, samples were fixed with 2% paraformaldehyde for 10 min at room temperature, followed by a 10-min incubation with methanol at −20 °C. Samples were incubated with primary antibody for 1 h or overnight, secondary antibody for at least 30 min, and mounted with ProLong Diamond Antifade Mountant (Invitrogen P36971). Images were acquired with a Zeiss AxioImager M2 microscope equipped with Apotome 2, Axiocam 702 mono camera, and Zeiss PLAN APOCHROMAT 63X/1.4NA Oil DIC objective. Samples stained with the same antibodies during the same experiment were captured using the same acquisition time. Analysis was performed using ImageJ/FIJI.

### Transmission electron microscopy

HeLa Parkin cells grown on coverslips made of Aclar film (Electron Microscopy Science) were fixed in 2.5% glutaraldehyde and 3.2% paraformaldehyde in 0.1 M sodium cacodylate buffer, pH 7.0 at room temperature for 2 min using a 250 W microwave (microwave biological sample preparation system, Pelco BioWave Pro+). After a 1-min buffer rinse for three times using a 250 W microwave, the samples were post fixed in 1% OsO_4_ in the same buffer for 2 min using a 100 W microwave at room temperature. After a 1-min buffer rinse three times using a 250 W microwave, samples were serially dehydrated in ethanol and propylene oxide before being infiltrated with Spurr’s resin and microwave-assisted embedding. The embedded samples were sectioned with a Leica Reichert Ultracut S or Leica EM UC6 ultramicrotome. Ultra-thin sections (∼90 nm) were stained with 5% uranyl acetate/50% methanol and 0.4% lead citrate/0.1 N NaOH. A FEI Tecnai G2 Spirit Twin TEM at 80 kV was used for viewing, and micrographs were captured with a Gatan Orius CCD camera.

### Mitophagy assays

The clearance of mitochondria was assessed by Western blot analysis of mitochondrial inner membrane protein COXIV, and by immunofluorescent quantitation of mitochondrial matrix protein ATP5B-positive puncta using microscopy followed by ImageJ analysis. For the assessment of mitophagy flux, a plasmid expressing 2xCOX8-EGFP-mCherry-PEST was either transiently transfected into the HeLa Parkin or SH-SY5Y cells with lipofectamine or TransIT-X2 Dynamic Delivery System (Mirus Bio) according to manufacturer’s instruction. The numbers of mitolysosomes (red-only puncta) were quantified with the ImageJ script (mQC counter) published by Dr Ian Ganley (52).

### Assessment of protein ubiquitylation

HeLa Parkin cells were transfected with 6xHis tagged ubiquitin-expressing construct by Lipofectamine 2000 (Thermo Fisher Scientific, 11668500) and treated with either DMSO or OA 4 h. Cell lysates were prepared by ultrasonication with denaturing Buffer A [6 M guanidine hydrochloride, 0.1 M Na_2_HPO_4_/NaH_2_PO_4_ (pH 8.0), and 10 mM imidazole] supplemented with cOmplete protease inhibitor cocktail, phosphatase inhibitor, and 10 mM NEM. Cell lysates (3 mg) were incubated at 4 °C overnight with 15 μl bed volume of Ni-NTA agarose prewashed with PBS. The beads were washed 5 min three times with a mixture of Buffer A and Buffer TI [25 mM Tris–HCl (pH 6.8) and 20 mM imidazole] at a ratio of 1:3, and five times with Buffer TI. The beads were resuspended with Laemmli buffer containing 5% β-mercaptoethanol, boiled at 95 °C for 10 min, and analyzed by Western blot.

### USP2 deubiquitinase assay

Cell lysates (1 mg) were immunoprecipitated with either anti-FLAG M2 antibody or Ubiquitin-Trap Agarose (ChromoTek #uta) and washed with the cell lysis buffer [25 mM Hepes (pH 7.4), 150 mM NaCl, 1 mM EDTA] for three times and washed once with USP2 reaction buffer [50 mM Tris–HCl (pH 7.5), 150 mM NaCl, and 1 mM DTT]. The washed samples were incubated with or without 20 pmol of the recombinant catalytic domain of USP2 (258-605 a.a.) (a gift from Dr Ta-Hsien Lin, National Yang Ming Chiao Tung University, Taiwan) in 50 μl of USP2 reaction buffer for 40 min prior to Western blot analysis.

### Statistical analysis

All statistical parameters and values are reported in the Figures and corresponding Figure Legends. For comparisons of group means, one-way ANOVA was employed. For nonparametric comparisons involving multiple groups—such as mitochondrial clearance and mitophagy flux assays—the Kruskal-Wallis test was used followed by Dunn’s test for multiple comparisons. For the OMM rupture biosensor analysis, ordinary One-way ANOVA with Holm-Sidak’s test was used for multiple comparison. The proportion of cells with mitochondrial localization of VCP, NDP52, and OPTN, as well as the frequency of ruptured *versus* unruptured mitochondria in the TEM study, were analyzed using the Chi-square test. All statistical tests were two-tailed.

## Data availability

All data are available in the main text and the supporting information.

## Supporting information

This article contains supporting information.

## Conflict of interest

The authors declare that they have no conflicts of interest with the contents of this article.
