# Supplementary material for: The ubiquitin-binding protein ANKRD13A mediates VCP-dependent mitochondrial outer membrane rupture during PINK1/Parkin-mediated mitophagy[image]
Source: J Biol Chem. 2025 Sep 18;301(11):110739. doi: 10.1016/j.jbc.2025.110739 (PMC12685520; doi:10.1016/j.jbc.2025.110739)
Supplement: Supplementary Figures [file mmc1.pdf]

Figure S1, Related to Figure 2

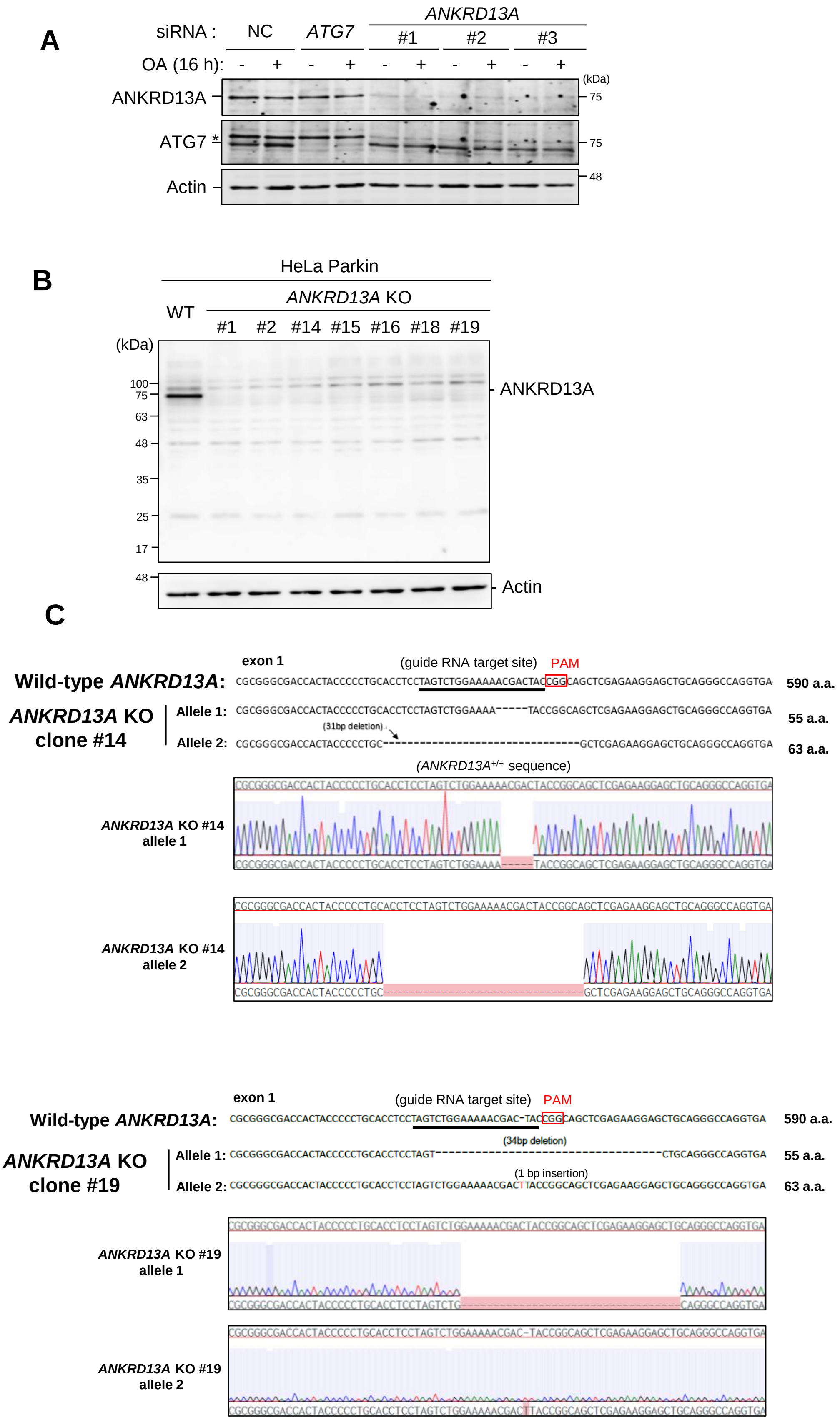

D

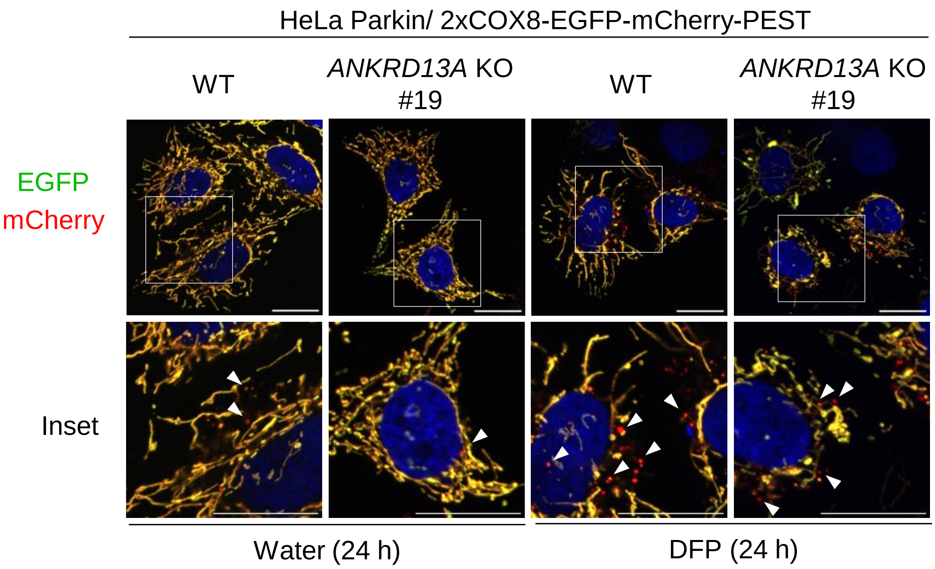
HeLa Parkin/ 2xCOX8-EGFP-mCherry-PEST
WT
ANKRD13A KO #19
WT
ANKRD13A KO #19
EGFP
mCherry
Inset
Water (24 h)
DFP (24 h)

E

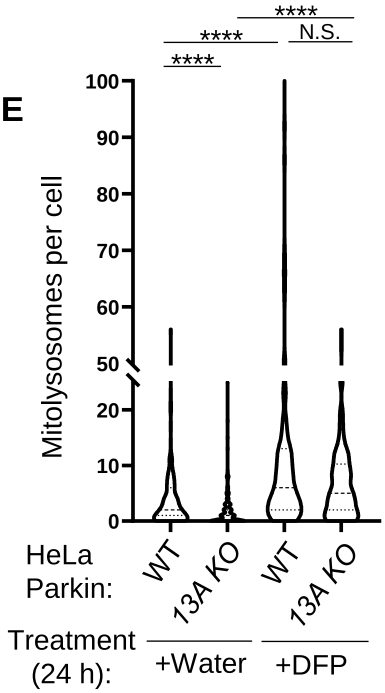
****
****
N.S.
****
Mitolysosomes per cell
100
90
80
70
60
50
20
10
0
HeLa Parkin:
WT
13A KO
WT
13A KO
Treatment (24 h):
+Water
+DFP

Figure S2, Related to Figure 5

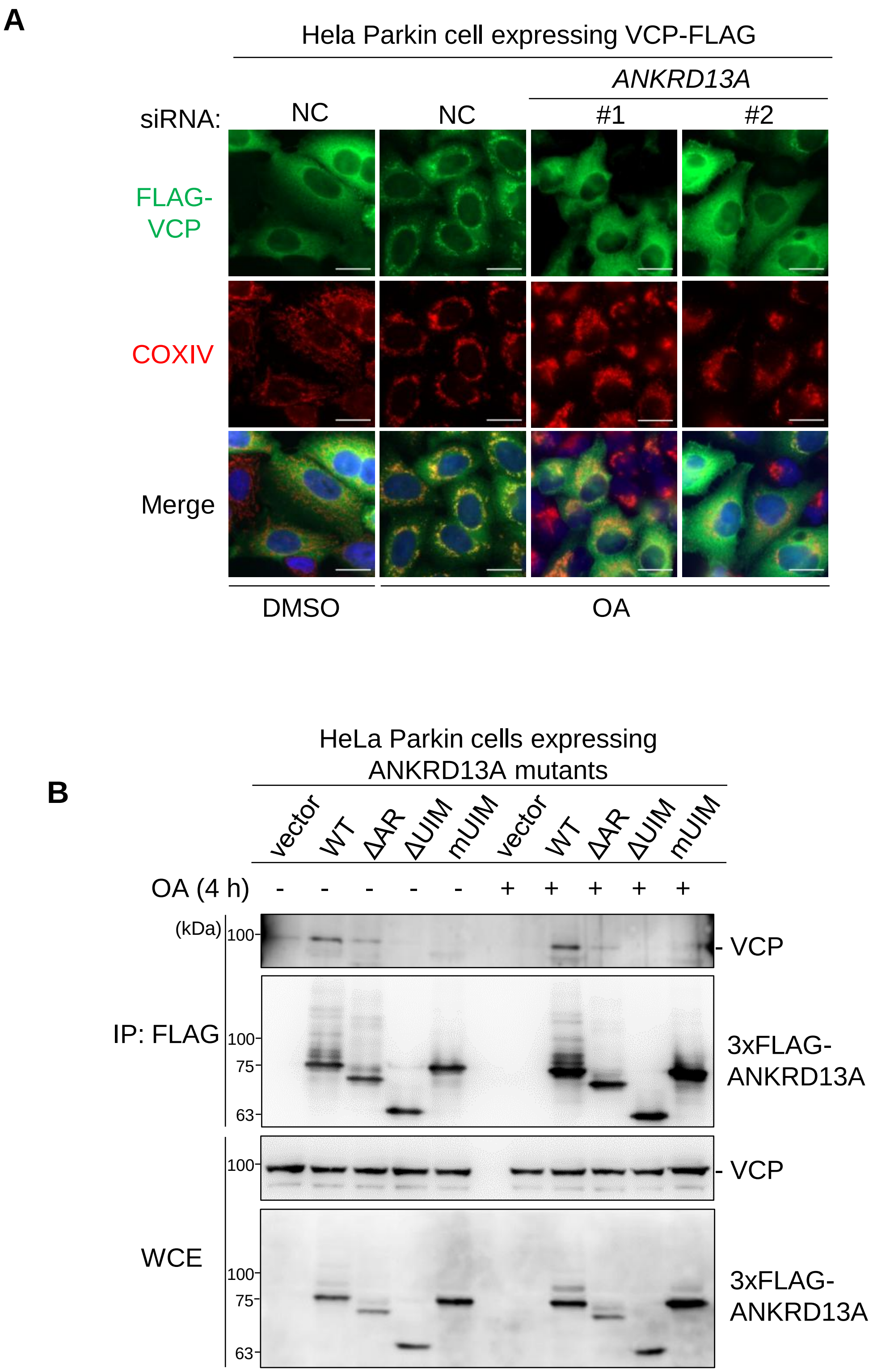

Figure S3, Related to Figure 6

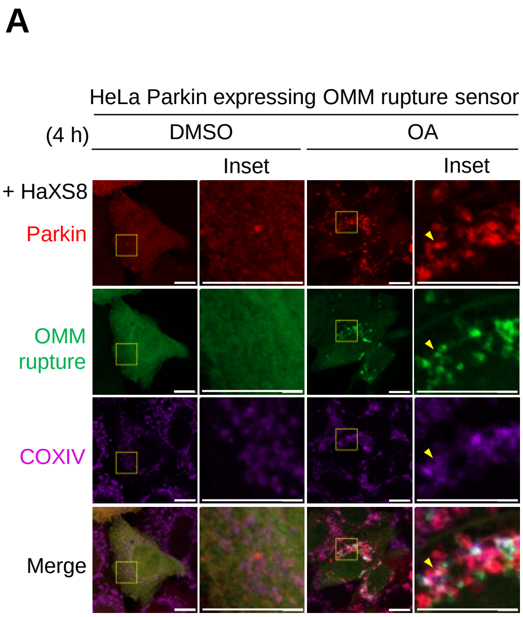

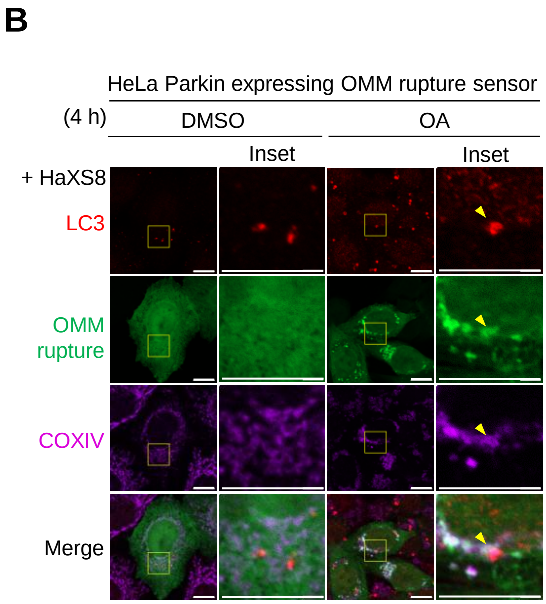

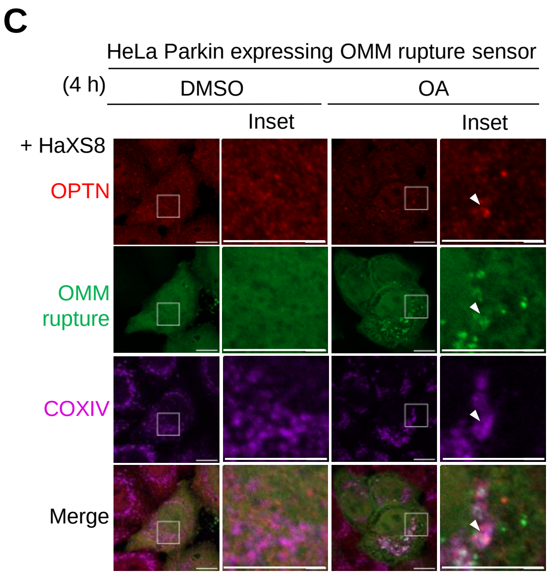

Figure S4, Related to Figure 7

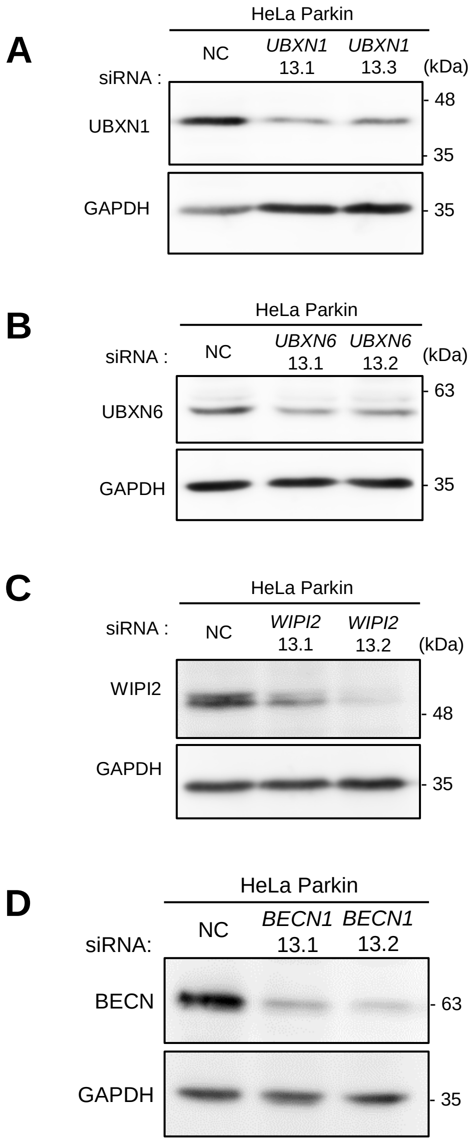

Figure S5

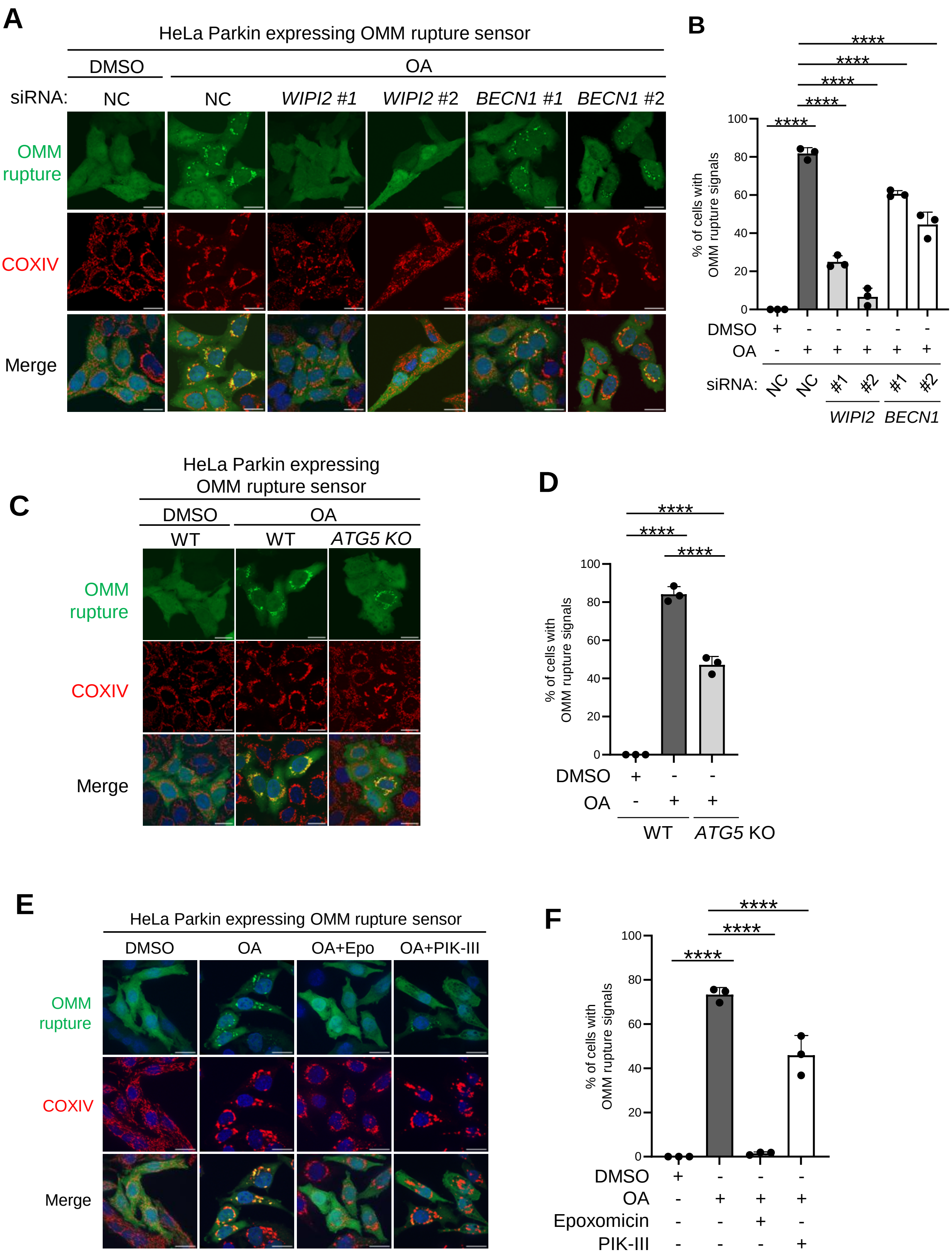
A
HeLa Parkin expressing OMM rupture sensor
DMSO
OA
siRNA:
NC
NC
WIPI2 #1
WIPI2 #2
BECN1 #1
BECN1 #2
OMM rupture
COXIV
Merge
B
% of cells with OMM rupture signals
****
DMSO + - - - - -
OA - + + + + +
siRNA: NC NC #1 #2 #1 #2
WIPI2
BECN1
C
HeLa Parkin expressing OMM rupture sensor
DMSO
OA
WT
WT
ATG5 KO
OMM rupture
COXIV
Merge
D
% of cells with OMM rupture signals
****
DMSO + - -
OA - + +
WT
ATG5 KO
E
HeLa Parkin expressing OMM rupture sensor
DMSO
OA
OA+Epo
OA+PIK-III
OMM rupture
COXIV
Merge
F
% of cells with OMM rupture signals
****
DMSO + - - -
OA - + + +
Epoxomicin - - + -
PIK-III - - - +

Figure S6

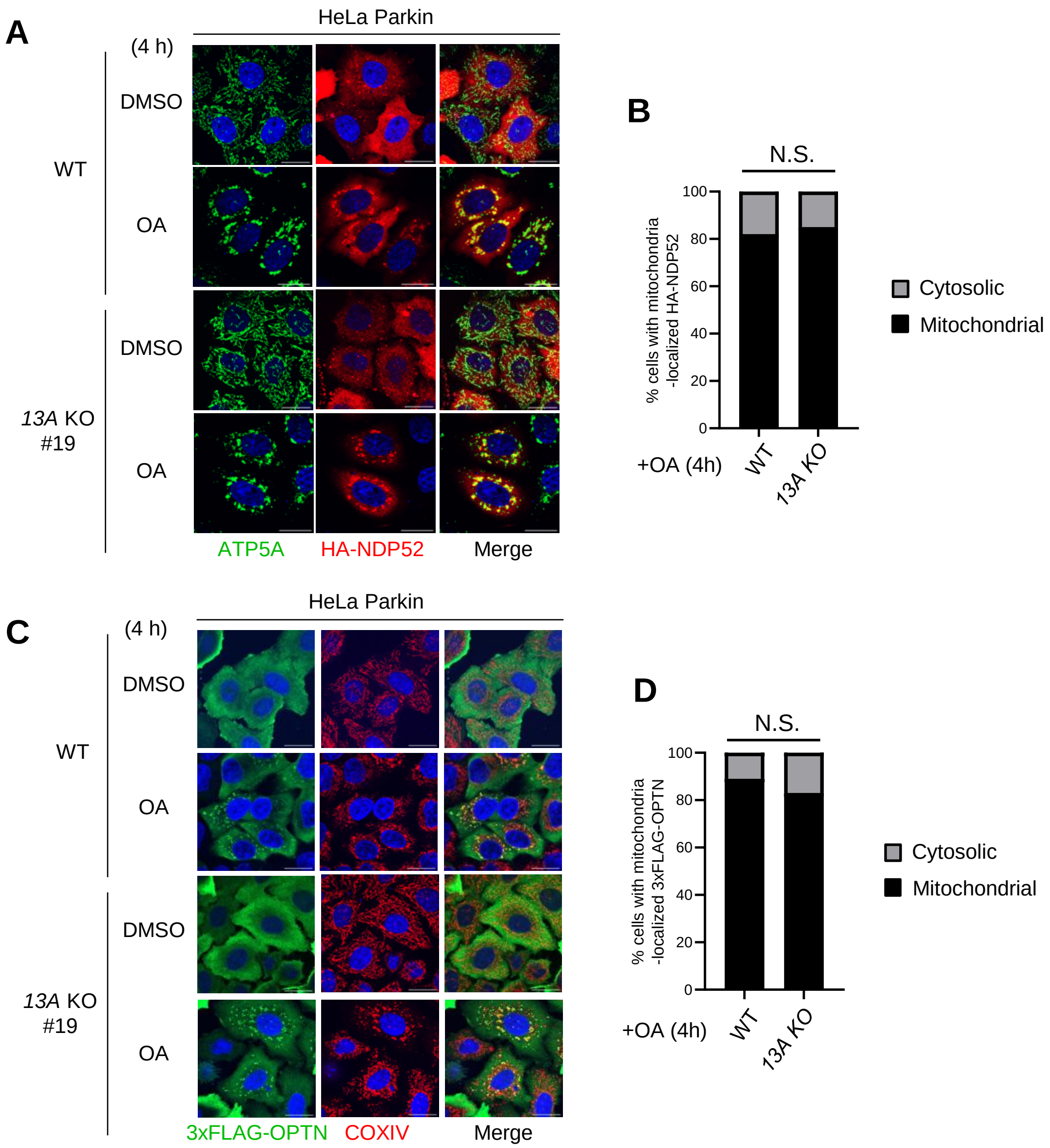
A
HeLa Parkin
(4 h)
DMSO
OA
DMSO
OA
WT
13A KO
#19
ATP5A
HA-NDP52
Merge
B
N.S.
% cells with mitochondria
-localized HA-NDP52
100
80
60
40
20
0
+OA (4h)
WT
13A KO
Cytosolic
Mitochondrial
C
HeLa Parkin
(4 h)
DMSO
OA
DMSO
OA
WT
13A KO
#19
3xFLAG-OPTN
COXIV
Merge
D
N.S.
% cells with mitochondria
-localized 3xFLAG-OPTN
100
80
60
40
20
0
+OA (4h)
WT
13A KO
Cytosolic
Mitochondrial

Figure S1 (related to Figure 2).

(A) Western blot analysis of ANKRD13A and ATG7 in HeLa Parkin cells treated with the indicated siRNA. Asterisks denote the signals of ANKRD13A from the previous blot. **(B)** Western blot to confirm the depletion of ANKRD13A in the candidate KO cell lines. **(C)** Genotyping of select *ANKRD13A* KO HeLa Parkin cells. Genomic sequence around the guide RNA target site was PCR-amplified and followed by TA cloning and Sanger sequencing. The chromatogram and alignment of *ANKRD13A* KO alleles revealed frameshift mutations in the *ANKRD13A* KO cell lines #14 and #19. **(D)** Representative immunofluorescent images of mitolysosomes of wild-type (WT) and *ANKRD13A* KO HeLa Parkin cells transiently transfected with mitophagy flux reporter (2xCOX8-EGFP-mCherry-PEST). Cells were treated with either water or 1 mM deferiprone (DFP) for 8 h prior to the analysis. Arrows indicate mitolysosomes (red). Scale bars, 20 μm. **(E)** Quantitation of mitolysosomes in (d). >120 cells analyzed per sample. ****$P<0.0001$, N.S. non-significant. One-way ANOVA with multiple comparisons.

Figure S2 (related to Figure 5).

(A) Representative immunofluorescent micrographs of mitochondrial recruitment of VCP during OA-induced mitophagy. HeLa Parkin cells expressing FLAG-tagged VCP were transfected with control (NC) or two different siRNA targeting *ANKRD13A*. The cells were treated with either DMSO or OA for 4 h and immunofluorescently stained with anti-FLAG (FLAG-VCP) or anti-ATP5B (mitochondrial marker) antibodies. Scale bars, 20 μm. **(B)** Co-immunoprecipitation of ANKRD13A mutants with VCP. HeLa Parkin *ANKRD13A* KO cells expressing empty vector (vector), wild-type (WT), or the indicated mutant ANKRD13A were treated with either DMSO or OA for 4 h. Samples were immunoprecipitated with anti-FLAG antibody and analyzed by Western blot as indicated.

Figure S3 (related to Figure 6).

The colocalization of OMM rupture signal with Parkin (a), LC3 (b), and OPTN (c). HeLa Parkin cells expressing the OMM rupture sensor were incubated with either DMSO or OA for 4 h and HaXS8 (500 nM) during the last hour of incubation, and samples were immunofluorescently stained with anti-Parkin and COXIV (mitochondria) antibodies. Arrows indicate the colocalization of Parkin, LC3, or OPTN with OMM rupture signals. Scale bars, 10 µm.

Figure S4 (related to Figure 7):

Knockdown efficiency of UBXN1 (a), UBXN6 (b), WIPI2 (c), and BECN1(d) in HeLa Parkin cells. Cells were treated with either non-targeted control (NC) or the indicated siRNA for 48 h prior to the Western blot analysis.

Figure S5:

The effects of genetic and pharmacological inhibition of autophagy on OA-induced OMM rupture. **(A)** Representative images of HeLa Parkin cells expressing the OMM rupture sensor subjected to control (NC), *WIPI2*, or *BECN1* siRNA knockdown. Cells were incubated with either DMSO or OA for 4 h and HaXS8 (500 nM) during the last hour of incubation. **(B)** Quantification of OMM rupture in (a). **(C)** Representative images of wild-type (WT) or *ATG5* KO HeLa Parkin cells expressing the OMM rupture sensor incubated with either DMSO/OA and HaXS8 as in (a). **(D)** Quantification of OMM rupture in (c). **(E)** Representative images showing HeLa Parkin cells expressing the OMM rupture sensor treated with either DMSO/OA and HaXS8 as described above. Cells were also co-treated with either PIK-III or Bafilomycin A1 (BafA1) during the incubation with OA. **(F)** Quantification of OMM rupture in (e). ****$P<0.0001$, One-way ANOVA with multiple comparisons. Scale bars, 20 µm.

Figure S6:

Mitochondrial recruitment of NDP52 and OPTN is not impaired upon the depletion of ANKRD13A. **(A, C)** HeLa Parkin wild-type (WT) or *ANKRD13A* KO (13A KO) cells expressing either HA-NDP52 (a) or 3xFLAG-OPTN (c) were treated with either DMSO or OA for 4 h prior to immunofluorescent staining with the indicated antibodies. ATP5A and COXIV, mitochondrial markers. Scale bars, 20 µm. **(B, D)** Quantification of mitochondria-localized NDP52 or OPTN. >100 cells analyzed per sample. Chi-square test, N.S. non-significant.
